# Feast to famine: Sympatric predators respond differently to seasonal prey scarcity on the low Arctic tundra

**DOI:** 10.1002/ece3.9951

**Published:** 2023-03-27

**Authors:** Chloé Warret Rodrigues, James D. Roth

**Affiliations:** ^1^ Department of Biological Sciences University of Manitoba Winnipeg Manitoba Canada

**Keywords:** climate change, movement ecology, range expansion, resource fluctuation, seasonality, telemetry

## Abstract

Resource fluctuation is a major driver of animal movement, influencing strategic choices such as residency vs nomadism, or social dynamics. The Arctic tundra is characterized by strong seasonality: Resources are abundant during the short summers but scarce in winters. Therefore, expansion of boreal‐forest species onto the tundra raises questions on how they cope with winter‐resource scarcity. We examined a recent incursion by red foxes (*Vulpes vulpes*) onto the coastal tundra of northern Manitoba, an area historically occupied by Arctic foxes (*Vulpes lagopus*) that lacks access to anthropogenic foods, and compared seasonal shifts in space use of the two species. We used 4 years of telemetry data following 8 red foxes and 11 Arctic foxes to test the hypothesis that the movement tactics of both species are primarily driven by temporal variability of resources. We also predicted that the harsh tundra conditions in winter would drive red foxes to disperse more often and maintain larger home ranges year‐round than Arctic foxes, which are adapted to this environment. Dispersal was the most frequent winter movement tactic in both fox species, despite its association with high mortality (winter mortality was 9.4 times higher in dispersers than residents). Red foxes consistently dispersed toward the boreal forest, whereas Arctic foxes primarily used sea ice to disperse. Home range size of red and Arctic foxes did not differ in summer, but resident red foxes substantially increased their home range size in winter, whereas home range size of resident Arctic foxes did not change seasonally. As climate changes, abiotic constraints on some species may relax, but associated declines in prey communities may lead to local extirpation of many predators, notably by favoring dispersal during resource scarcity.

## Graphical abstract

Edge populations usually face limiting biotic or abiotic conditions, which may shift with environmental change. To examine how expanding species cope with the strong resource fluctuation in the tundra in comparison with native species, we compared space use of red and Arctic foxes between seasons of resource plenty and scarcity. Most foxes dispersed when resources became scarce, despite high mortality risks, but only red foxes increased their home range size when they stayed resident during food scarcity. As climate changes, abiotic constraints on some species may relax, but associated prey declines may trigger dispersal of others, eventually leading to local extirpation.

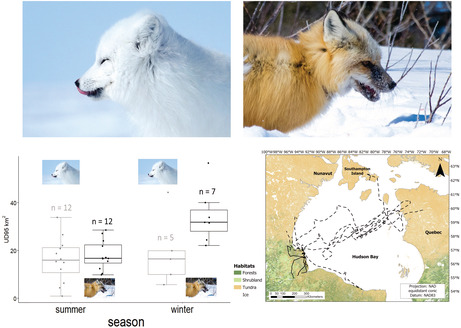

## 1 INTRODUCTION

Species’ range expansions rank among the preeminent ecological consequences of Arctic warming and anthropogenic pressure (McCarty, 2001). For example, species that primarily inhabit the boreal forest have settled onto the tundra due to milder winters, longer productive periods, and increased availability of anthropogenic subsidies (Gallant et al., 2020; Hersteinsson & Macdonald, 1992; Tape et al., 2016). However, at the edge of its distribution, a species also reaches the limits of its environmental tolerances (biotic and abiotic). Scarce patches of preferred habitat, lower resource availability, and harsh winters may challenge the survival of peripheral individuals and force them to adjust their behavior, including ranging behavior (e.g., Linnell et al., 2021; Niedzielski & Bowman, 2016).

Movement tactics are driven by ecological, social, and morpho‐physiological factors acting in synergy, such as resource availability, body size, seasonality, or the distribution of prey or competitors (Macdonald & Johnson, 2015). Although most individuals restrict their ranging behavior to familiar areas to meet their needs efficiently, some can disperse if the cost of staying in an area exceeds the benefits (e.g., Avgar et al., 2014). Individuals usually favor residency when they find abundant and predictable resources and can avoid competitors or predators (Jonzén et al., 2011; Marneweck et al., 2019). Residents’ successive maintenance movements (i.e., movements performed within the context of necessary activities to survive and reproduce; Roshier & Reid, 2003), as measured over short time periods, typically occur over relatively short distances and do not produce a net displacement along a movement vector over a longer time period. The succession of these maintenance movements thus perpetuates a home range (or a territory if actively defended) (e.g., Powell, 2000). In contrast, individuals may engage in long‐range movements when they cannot predict resource availability, nor avoid adverse weather conditions, competitors or predators (Hsiung et al., 2018; Jonzén et al., 2011). Long‐range movements occur on a continuum. Unlike migrations, nomadic movements lack directionality and regular timing: The animal leaves its former range permanently and may wander over long time periods (Roshier & Reid, 2003). Although carnivores typically exhibit residency, they may engage in long‐range movements to reproduce or settle in more suitable habitat, resulting in dispersal (Roshier & Reid, 2003).

Resource availability influences not only movement tactic and ranging behavior (and hence home range sizes) but also the degree to which competitors tolerate each other (Eide et al., 2004; Maher & Lott, 2000; Mcloughlin et al., 2000). The resource dispersion hypothesis predicts that home range size increases with increased resource dispersion, while territoriality decreases with increased food abundance. When resources are highly unpredictable, an individual (or breeding pair) will likely maintain a home range large enough to meet its needs during times of scarcity (Macdonald, 1983). Furthermore, Maher and Lott (2000) hypothesized that as resource predictability decreases, so does the net benefit of territoriality, except in food‐caching species, which still benefit from being territorial. This territorial benefit was empirically corroborated in fieldfares (*Turdus pilaris*), which defend stored food in anticipation of food scarcity, and in Arctic foxes (*Vulpes lagopus*), which defend food caches and exhibit the lowest home range overlap in areas where prey are unpredictable (Eide et al., 2004; Maher & Lott, 2000).

Arctic ecosystems are characterized by marked seasonality and interannual resource fluctuation (Jonzén et al., 2011; Korpimäki & Hongell, 1986). Low availability of resources in winter contrasts with a summer resource burst; geese, seabirds, and shorebirds reproduce every summer in the Arctic, offering an abundant and predictable food source to predators, if only for a limited period (Eide et al., 2004; McDonald et al., 2017; Tannerfeldt & Angerbjörn, 1998). Many Arctic predators primarily rely on arvicoline rodents (lemmings and voles) that are present year‐round but whose fluctuating populations peak every 3–4 years (Fauteux et al., 2015; Krebs et al., 2002). Together, rodent abundance fluctuations and the relatively short lifespan of mammalian predators make rodents an unpredictable resource (Bilodeau et al., 2013; Krebs et al., 2002; Tannerfeldt & Angerbjörn, 1998).

Predators may thus migrate or disperse, either to track their preferred prey (Jonzén et al., 2011; Korpimäki & Hongell, 1986) or because peaks of rodent abundance have favored a higher consumer density, which reduces per capita energy intake (Mysterud et al., 2011) when rodent abundance decreases again (Avgar et al., 2014; Robillard et al., 2016). In that context, long‐range movement may be an adaptive tactic to reduce competition between consumers or alleviate the negative effects of food scarcity on survival and reproduction. However, such movements are often associated with high rates of mortality because individuals lack familiarity with or adaptation to the landscapes they cross (Korpimäki & Hongell, 1986; Powell & Mitchell, 2012; Roth, 2003). Therefore, terrestrial predators typically favor residency (Lai et al., 2017; Powell, 2012) and develop strategies to cope with prey scarcity while retaining their home ranges. Examples of such strategies include demographic lability (Barraquand & Benhamou, 2008), food caching (Sklepkovych & Montevecchi, 1996), larger home range maintenance to cope with prey scarcity (Eide et al., 2004), and increased frequency of short extraterritorial trips (excursions) to exploit alternative resources (Lai et al., 2017; Messier, 1985).

The harsh Arctic conditions historically limited the northern distribution of red foxes (*Vulpes vulpes*; Hersteinsson & Macdonald, 1992; Bartoń & Zalewski, 2007; Gallant et al., 2020), but during the 20th century, red foxes considerably extended their range into the Arctic due to increased availability of anthropogenic subsidies that buffered winter food scarcity in many Arctic areas (Gallant et al., 2020). Red and Arctic foxes are ecologically similar: They use dens to reproduce and raise their young, beginning shortly before migratory birds arrive, and although they depend strongly on arvicoline rodents, they forage opportunistically and cache food (Careau, Giroux, & Berteaux, 2007; McDonald et al., 2017; Roth, 2002). However, red foxes are larger than Arctic foxes, which increases their food requirements (Carbone et al., 2007), and are less adapted than Arctic foxes to prey scarcity during the harsh Arctic winters (Careau, Morand‐Ferron, & Thomas, 2007; Fuglesteg et al., 2006).

We examined movement tactics and space use by red and Arctic foxes on the low Arctic tundra in northern Manitoba, Canada, where red foxes recently expanded from the adjacent boreal forest and now reproduce in sympatry with Arctic foxes (Moizan et al. (2023), submitted; Zhao et al., 2022). Seasonal variability of resources likely drives movement tactics in both red and Arctic foxes. In that context, we hypothesized that winter conditions are limiting for red foxes, in contrast to Arctic foxes and compared with summer. Specifically, red foxes are evolutionarily rooted in the boreal forest (Kamler & Ballard, 2002; Wells & Aubry, 2011) and, thus, lack adaptations to exploit the sea ice (Colson et al., 2017; Klein & Sowls, 2015). In addition, their increased energetic requirements during winter (Fuglesteg et al., 2006) will likely constrain their ranging behavior. We thus predicted that long‐range movements are primarily initiated during winter (P1), red foxes are more likely to disperse in search of better conditions instead of commuting to the sea ice and back like Arctic foxes do (P2) due to their larger size and higher energetic needs, red foxes always maintain larger home ranges than Arctic foxes (P3), extraterritorial excursions are more frequent in winter in both species (P4), and extraterritorial excursions occur more frequently for resident red foxes than for Arctic foxes (P5).

## 2 MATERIALS AND METHODS

### 2.1 Study area and species

**FIGURE 1:**
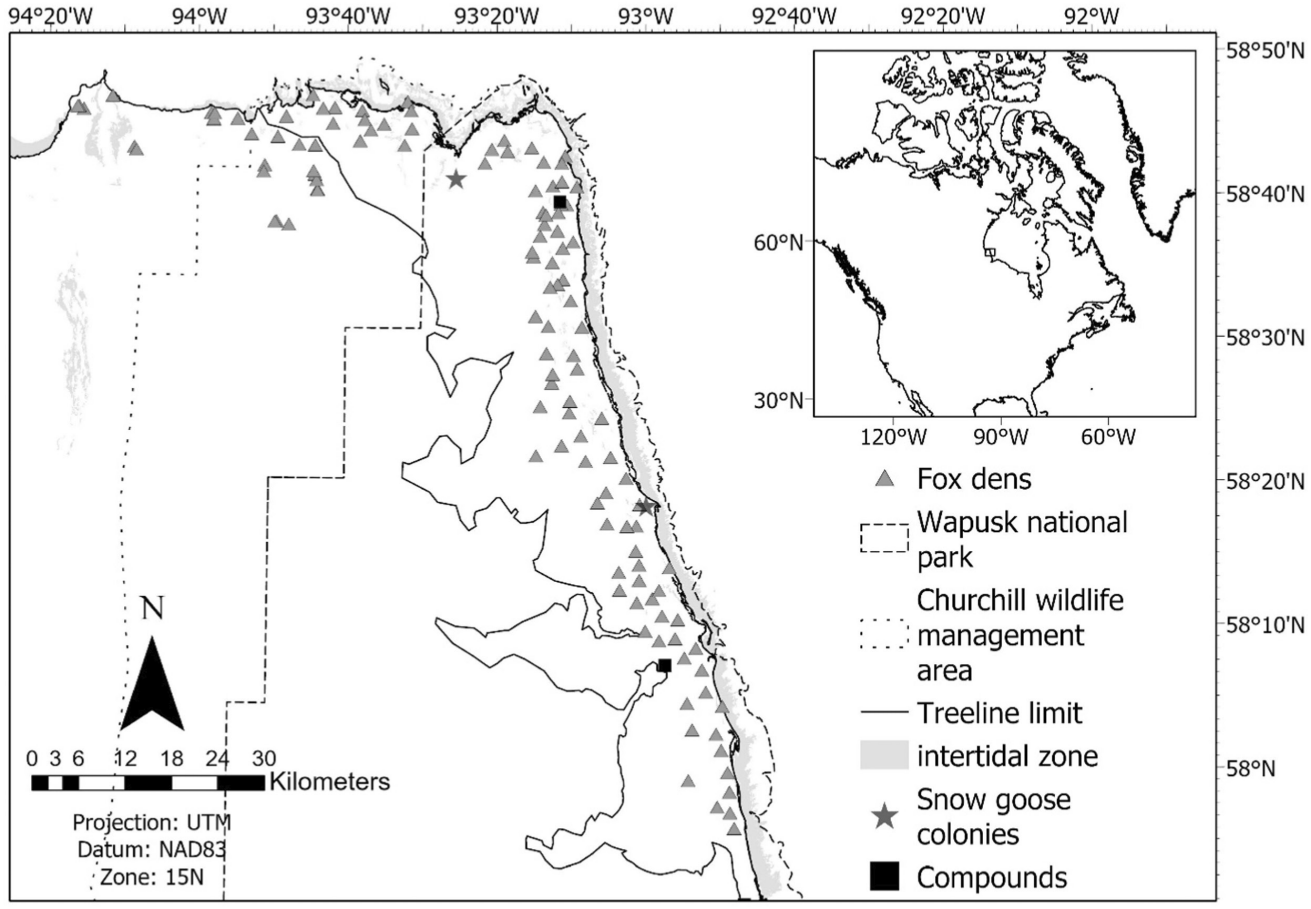
Map of our study area in northern Manitoba, Canada.

Our study area near Churchill, Manitoba (Figure 1; 58° N, 94° W), is part of the Hudson Bay Lowlands, a uniformly flat (<200 m elevation) wetland bordering the southwestern shore of Hudson Bay (Brook & Kenkel, 2002). This wet tundra ecosystem lies between the boreal forest to the south and west and the marine ecosystem to the north and east. The three biomes thus transition in our study area. In fall, this part of Hudson Bay freezes as early as the first week of November, and the ice along the northern and western coasts of the Bay is typically consolidated by December 2, providing a platform for fox movements and opportunities to forage on marine resources. Sea ice in the area breaks up around mid‐June, and the area is typically free of ice by the first week of July (Hochheim et al., 2010), thus limiting access to marine resources on the sea ice until the ocean freezes again. We considered that the sea ice starts at the low tideline (Ponomarenko et al., 2014).

Lemmings are available year‐round, but their peak abundance has dramatically declined throughout the Arctic (e.g., Bilodeau et al., 2013), particularly for low Arctic populations that are sympatric with voles (Ehrich et al., 2020). Abundant populations of Canada geese (*Branta canadensis*) and lesser snow geese (*Anser caerulescens*) nest each year in the study area, providing an important food source to predators (McDonald et al., 2017). Canada goose nests are distributed throughout the entire area along with some snow goose nests, and two major snow goose colonies (>20,000 nesting pairs) occur near the coast (Figure 1; Andersen et al., 2010; McDonald et al., 2017). Peak arrival of snow geese occurs during the first week of May (Cargill & Jefferies, 1984) and >95% of Canada goose nests are initiated before the last week of May, with a median hatch date during the 3rd week of June (Andersen et al., 2010). Geese remain abundant throughout fall and may be present until late October—the latest observation of snow or Canada geese in the Churchill area based on band recoveries for the 2017–2019 period was on October 16 (Celis‐Murillo et al., 2020).

*Capture and satellite telemetry—*Between 2017 and 2019, we captured 10 red foxes and 13 Arctic foxes using Tomahawk (Model 208, Tomahawk Live Trap Co., WI) and padded leghold traps (Softcatch # 1.5, Oneida Victor Ltd, USA). Traps were placed on active dens or by protruding features (e.g., driftwood or spruce islets) and remained open continuously for up to one week. We checked the traps every 4–6 h and closed them during extreme weather conditions (e.g., blizzard or temperatures below −25°C). We captured adult foxes from March to May when snow still covered the ground and facilitated travel over large distances, except for two adult foxes caught near our field camps in June 2018. We did not anesthetize the foxes, which were easily handled without chemical restraint. Foxes were first wrapped in a blanket and released from the traps; then, we assessed sex and body condition, deployed an Iridium satellite collar (#4170 or 4270, Telonics, Mesa, Arizona, USA; ~100 g, that is, 2%–4% of a fox body mass), and released them at the site of capture. All handling procedures were approved by the University of Manitoba Animal Care Committee (Protocol F17‐012), and the research was conducted under Parks Canada Research and Collection Permits WAP‐2017‐25,781 and WAP‐2018‐27,938, and Manitoba Wildlife Scientific Permits WB20226 and WB21856.

### 2.2 Movement analysis

Our GPS collars used different schedules throughout the year (see Table S1), so we thinned all the tracks by randomly selecting 1 location per day (the lowest fix frequency). We defined two relevant contrasting periods based on goose phenology. The season of abundant resources (hereafter summer) thus extended from May 15, the approximate date of nest initiation, to the end of October, the last month during which geese can be considered alternative prey for the foxes of this area (Andersen et al., 2010; McDonald et al., 2017). The resource‐scarcity period (hereafter winter) extended from November 1–May 14, when geese are absent and foxes mostly rely on arvicoline rodents.

We plotted all fox tracks in ArcGIS 10.3 (ESRI, 2017, Redland, CA, USA) to remove possible major erroneous locations and identify movement strategies: residency and long‐range movements. We labeled a fox as a resident only if it maintained a home range (i.e., showed nondirectional movements within a geographically circumscribed area) from the start of a given season until the end of that season or until its death, if it occurred after the area resulting from movement analysis had reached an asymptote. We never included the season of capture in movement tactic and home range comparisons, since we could not know if a fox was dispersing earlier that season. Using a subset of 16 individuals with 111–187 locations each, we determined that home range areas reached an asymptote with 38 locations on average. All our resident foxes exceeded this threshold with at least 61 locations. All foxes that underwent long‐range movements (hereafter dispersals) were considered dispersers, since none returned to their departure area (they either died dispersing or settled elsewhere). The dispersal events we used to compare movement tactics were not natal dispersal because we only included adults (at least 1.5 years old), unlike the track descriptions, which included all available tracks.

For each dispersal (including those initiated during the season of capture that were not included in any other analysis), we calculated the cumulative distance traveled (i.e., sum of straight‐line distances between successive daily relocations), the duration (starting with the last position within the home range boundaries), the cumulative to straight‐line distance ratio (a proxy for fox behavior during dispersal), the cardinal direction (the angle of the vector between first and last locations, degrees from due North), the main substrate used for movement (sea ice or land), and the average daily speed. We considered that the dispersal started with the last location in a home range prior to dispersal initiation, or at the point of capture if a fox did not exhibit residency prior to dispersal (and thus was likely captured while already dispersing), and ended with the first location associated with a settlement of >7 days in a new delimited ranging area (on land, not ice) or with the death of the fox. Although foxes can exhibit staged dispersal, exploring delimited areas for a temporary period ranging from a few days up to a few weeks (e.g., Walton et al., 2018), we never observed clear staging behavior.

We estimated residents’ home ranges and core areas, defined as the 95% and the 50% utilization distribution isopleths, respectively, with local convex hulls (LoCoH) using the package T‐LoCoH v.1.40.07 in R (Lyons et al., 2013). LoCoH are nonparametric estimates of utilization distributions and perform better than parametric kernel methods to identify boundaries (such as coastlines) and unused areas (Getz et al., 2007; Stark et al., 2017). As such, they are well‐suited for our main objective to determine if red foxes were using the sea ice. We were not specifically interested in the temporal partition of space within seasons since we modeled space use using only one location per day. We, therefore, set the user‐defined parameter *s* to 0, which entailed that the time‐scaled distance was equivalent to the Euclidian distance (Getz et al., 2007). Due to heterogeneous location densities, we used the adaptive method (a‐LoCoH). We selected the *a* value for each animal using the graph tools provided in the T‐LoCoH package and following the recommendations to minimize the risks of both excluding used areas and including unused areas. To estimate seasonal home range shifts in each fox, we measured summer and winter home range overlaps using the package T‐LoCoH.dev v. 1.34.00/r12 and the distance between their centroids estimated in ArcGIS 10.3 (ESRI, 2017). All home ranges and core areas are displayed in Supplemental file S1. Based on the same dataset, we also estimated home ranges (95% utilization distribution) using a classic bivariate kernel density estimator (KDE) with a reference bandwidth, with R package adehabitatHR v.0.4.20 (Calenge, 2006). Although we decided not to use kernel density methods in this study, we provide the areas resulting from the KDE in Table S2, for comparison purposes.

Many residents undertook short‐distance and short‐duration trips outside the boundaries of their home range, either on land or on the sea ice. We defined excursions as any exploratory movement <7 days unusually far away from the current center of activity followed by a return to the home range. Home range borders include areas that are already peripheral to the center of activity. Therefore, to avoid making arbitrary decisions on a distance threshold to the border, we differentiated excursions from other movements near the home range border, based on the distribution of the distances between a location and the home range centroid. Locations that appeared to be outliers using a one‐sided Hampel filter (upper bound = median (Tukey‐transformed distance) + 3 median absolute deviations) were considered excursions. If a trip outside the boundaries of the LoCoH home range estimate consisted of multiple consecutive locations, we used the farthest away of the consecutive locations to determine if that trip was an excursion. Finally, we called “commuting trip” any excursion on the sea ice (Lai et al., 2017).

### 2.3 Statistical analyses

We performed all statistical analyses in R software (R Core Team, 2020). To compare the frequency of dispersal events (P1, P2) and home range size (P3) between species and between seasons, we used generalized linear mixed‐effect models (GLMM), family binomial (link logit) and Gaussian (link identity), respectively, using the lme4 package v.1.1–25 (Bates et al., 2015). We first checked the home range size data using fitdistrplus v.1.1.8 (Delignette‐Muller & Dutang, 2015) to assess which distributions fit our data the best, and both normal and gamma distribution were considered best fit according to the goodness‐of‐fit plots. We, thus, compared models with a gamma family, Gaussian/link identity and Gaussian/log link using AICc, and the Gaussian/identity model had by far the best AICc (i.e., 252.79 compared to 279.15 and 312.14 for the gamma and Gaussian/log link, respectively). We included species, season, and their interaction term and controlled for possible pseudo‐replication, using fox ID as a random effect. We reviewed three potential outlier foxes with leverage higher than 0.5 (Cooks’ distance) individually to decide whether they should be removed. Two red foxes settled in forest habitat after dispersing to the boreal forest near Gillam (Manitoba), which has a completely different habitat, prey abundance, diversity, and climate, which would strongly impact fox spatial behavior. Since the model cannot control for this source of variance based on two data points, we excluded these two outliers. We found no valid ecological or methodological reason to remove the outlying Arctic fox and thus retained that estimate in the data set. We assessed whether dispersal track parameters differed between species using two‐sided permutation tests based on the t statistics (n_perm_ = 9999) in library RVAideMemoire v. 0.9–79 (Hervé, 2021). We assessed the impact of dispersing on winter survival and annual survival using a right‐censored Cox mixed‐effect model from the coxme library (Therneau, 2020), controlling for fox ID as a random effect, and with time‐to‐event as the number of days since the start of a given winter (1 November). We then tested whether land excursions by resident foxes were more likely in winter and in red foxes (P4 and P5) using GLMMs to control for fox ID as a random effect: We transformed the raw number of excursions into a frequency of excursions per week, due to substantial interindividual variation in tracking period length. We checked our models’ assumption by plotting residuals versus fitted values, and the residuals showed no pattern. All summary statistics are presented as mean ± SE and/or mean [range] unless stated otherwise. Given our low sample size and individual heterogeneity in spatial behavior, we used an alpha threshold at 0.1 to lower the risk of type II error (e.g., Altman & Bland, 1995; Knaub, 1987).

## 3 RESULTS

We tracked 13 Arctic foxes and 10 red foxes between May 2017 and August 2020 (see Table S2 for capture details), which yielded a total of 6159 locations after thinning their tracks to one daily location, with 10 Arctic and 7 red foxes yielding enough data to perform home range analyses and assess seasonal shifts in space use. Since we followed 7 individuals for more than 1 year, we obtained 8 Arctic and 9 red fox home ranges over 3 winters, and 13 Arctic and 12 red fox home ranges over 4 summers. After thinning the tracks, the average time interval between 2 relocations was 26.19 ± 0.38 hours [2–312] (see detail for each track in Table S3).

### 3.1 Dispersal events

We recorded 14 dispersal events overall: 9 by Arctic foxes (8 individuals, since one fox dispersed twice) and 5 by red foxes (see Tables S4 and S5). One Arctic fox and one red fox were captured while dispersing (i.e., they were not using a home range at the time of capture and thus were excluded from all analyses beside dispersal track characterization) and 11 of the other 12 dispersals were initiated during winter (specifically between November 14 and May 10), while one was initiated on September 15 by an Arctic fox. Six of the eight Arctic and three of the five red foxes settled in a new area (at least temporarily, red foxes in forest and Arctic foxes in tundra habitats) after the dispersal events, but four Arctic foxes died 11 days–4 months after dispersing (at least one Arctic fox was caught by a fur trapper) and the three red foxes died 19 days–2 months after dispersing. Two red and two Arctic foxes died while dispersing. The two red foxes were caught by fur trappers, but we have no information on the cause of death for the two Arctic foxes.

**FIGURE 2:**
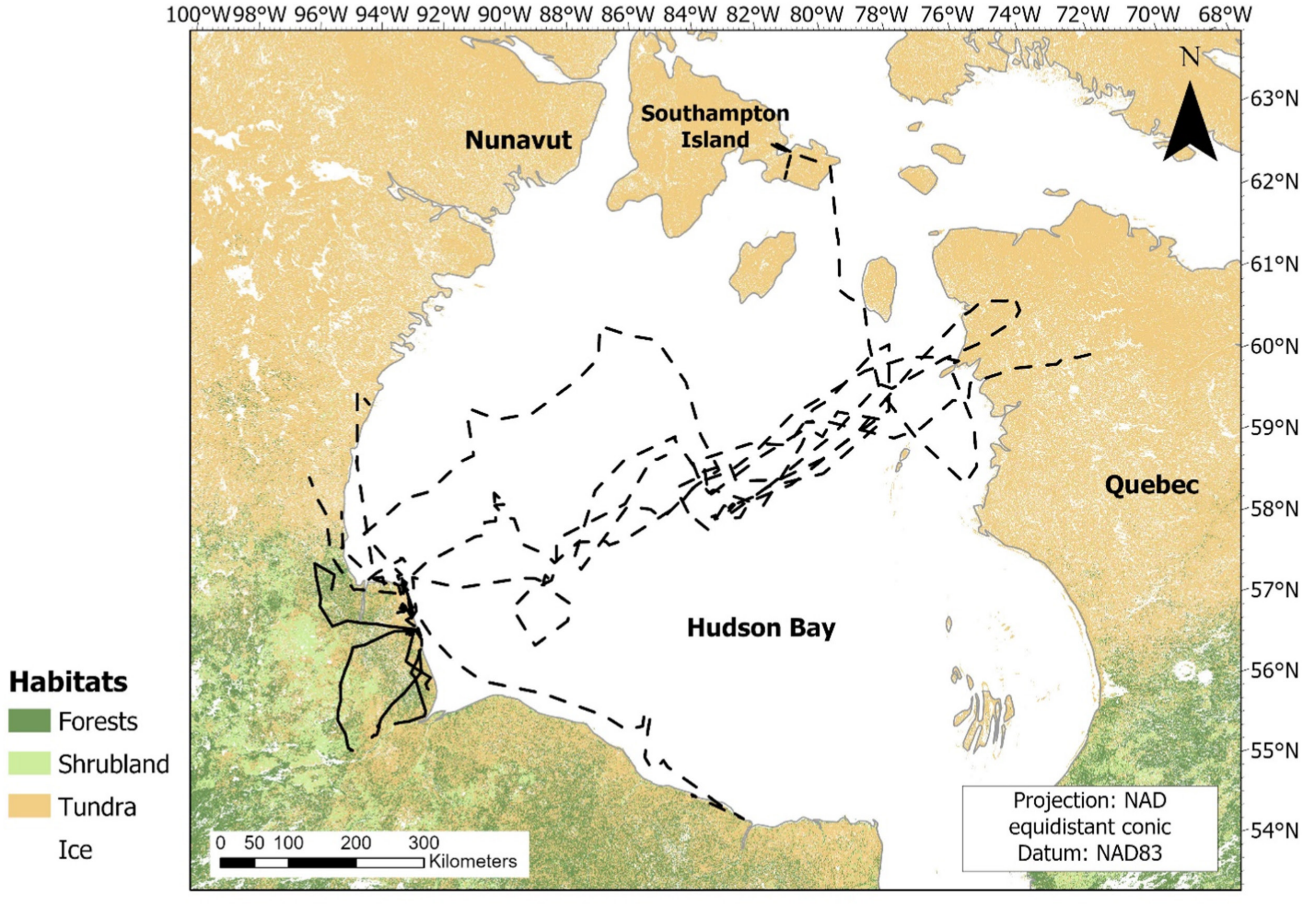
Dispersal tracks of red (solid lines) and Arctic (dashed lines) foxes captured in northeastern Manitoba, Canada, from 2017–2019.

All red foxes that dispersed moved toward forested areas, and all but one in a southwestern direction, whereas Arctic foxes moved toward other tundra habitats, eight to the northeast and northwest, and one to the southeast (but still in the Hudson Bay Lowlands; Figure 2). Of the nine Arctic foxes that dispersed, three used sea ice exclusively until they died or reached a new delimited ranging area, three navigated between sea ice and land, and three used land exclusively. No red foxes dispersed using sea ice. The mean length, duration, speed, and cumulative‐distance to straight‐line ratio of dispersal tracks were all smaller in red foxes, indicating they dispersed a shorter distance (permutation test: *t* = 1.44, *p* = .002, n_Arctic_ = 9, n_red_ = 5), over less time (*t* = 1.55, *p* = .036), with a slower speed (*t* = 1.78, *p* = .043), and more directly (*t* = 1.08, *p* = .020) than Arctic foxes (Table 1).

**TABLE 1:** Dispersal track parameters for each fox species, indicating different behaviors while dispersing.

Species	Parameter	Mean	se	Median	Min	Max	*n*
Red fox	Duration^a^	15.40	2.94	15.00	8.00	23.00	5
	Distance^b^	200.80	28.81	167.90	144.80	280.60	5
	CSLD ratio^c^	1.50	0.16	1.31	1.17	2.03	5
	Speed^d^	15.24	3.74	12.38	7.30	28.06	5
Arctic fox	Duration^a^	43.11	12.96	29.00	6.00	135.00	9
	Distance^b^	1243.00	529.60	781.70	216.30	5197.27	9
	CSLD ratio^c^	4.36	1.95	1.90	1.26	19.62	9
	Speed^d^	27.96	4.83	28.69	9.63	55.58	9

^a^ Days between start and end points of dispersal.

^b^ Sum of distances between successive relocations of dispersal track (cumulative distance, in km).

^c^ Ratio of cumulative to straight‐line distance (distance between start and end points of dispersal).

^d^ Average daily speed (km/day).

Of the 16 fox observations over three winters, 56% dispersed (5/9 red foxes and 5/7 Arctic foxes), but red foxes did not disperse more often than Arctic foxes (GLMM: *z* = −1.06, *p* = .29, *n* = 16). Dispersals were associated with higher mortality than residency: mortality risk was 9.4 times higher for dispersing foxes in the short‐term (i.e., during the same winter: *z* = −1.99, *p* = .046; n_disperse_ = 14, n_resident_ = 7) and 6.5 times higher in the long‐term (i.e., within a year of dispersing: *z* = −1.95, *p* = .051). However, mortality risk did not differ by species in the winter of dispersal (*z* = 1.29, *p* = .20; n_red_ = 10, n_Arctic_ = 11) or the following year (*z* = 0.42, *p* = .67).

### 3.2 Seasonal home range

**FIGURE 3:**
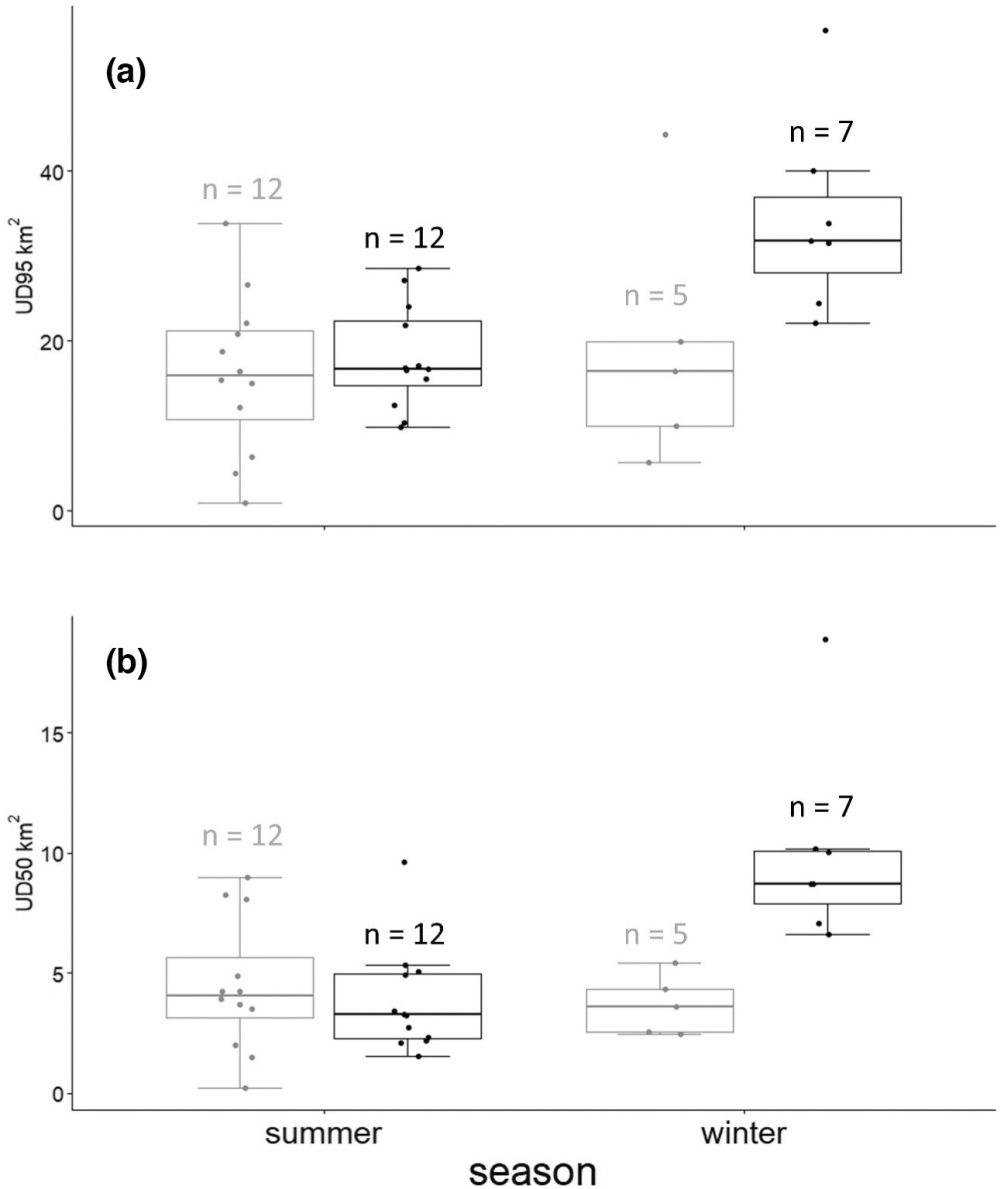
Size of (a) home ranges and (b) core areas of Arctic (gray) and red (black) foxes in northern Manitoba, Canada, in summer (n_red_ = 12, n_Arctic_ = 12) and winter (n_red_ = 7, n_Arctic_ = 5).

Resident fox space‐use patterns differed between species (Table 2). While summer home ranges of red foxes and Arctic foxes were similar, red foxes drastically increased the size of their home range in winter (Figure 3; GLMM: t_season_ = 0.72, *p* = .48, n_summer_ = 24, n_winter_ = 12; t_species_ = 0.14, *p* = .89, n_red_ = 19, n_Arctic_ = 17; t_species*season_ = 3.06, *p* = .006, n_total_ = 36). Winter home range and core areas of red foxes were 1.9 and 2.7 times larger than their summer home ranges and core areas, respectively. Two of the collared red foxes were a mated pair and like fox pairs elsewhere in the Arctic (Lai et al., 2022; Rioux et al., 2017), their home range sizes were similar (paired permutation t‐tests, home range: *t* = 9.78, *p* = .25; core area: *t* = 11.00, *p* = .25, *n* = 3).

**TABLE 2:** Home range (Utilization Distribution 95) and core area (UD 50) sizes (km^2^) per season and species.

Season	Species	UD	Mean	se	Min	Max	*n*
summer	Arctic fox	95	15.86	2.65	0.94^a^	33.91	12
winter	Arctic fox	95	19.81	6.47	8.59	44.32	5
summer	Red fox	95	18.06	1.77	9.84	28.61	12
winter	Red fox	95	34.72	4.17	23.90	56.58	7
summer	Arctic fox	50	4.03	0.68	0.22^a^	8.27	12
winter	Arctic fox	50	3.70	0.54	2.44	5.42	5
summer	Red fox	50	3.80	0.64	1.55	9.61	12
winter	Red fox	50	10.18	1.61	6.51	18.91	7

^a^ This fox had settled in a snow goose colony.

Individuals’ summer and winter home ranges overlapped moderately in both species, although the seasonal change in home range geometry was small for some (red foxes: 55.8 ± 11.9% [33.2%–70.7%], *n* = 7; Arctic foxes: 62.3 ± 12.3% [45.9%–75.4%], *n* = 4). The distance between winter and summer home range centroids was also relatively short (red foxes: 0.9 ± 0.6 km [0.4–2.0 km], *n* = 7; Arctic foxes: 0.8 ± 0.4 km [0.3–1.1 km], *n* = 4). Core areas, however, generally overlapped only slightly to moderately between seasons (red foxes: 21.4 ± 20.2% [0%–55.7%], *n* = 7; Arctic foxes: 29.9 ± 29.3% [1.5%–70.7%], *n* = 4), and so, the seasonal shift of core area centroids was often sizeable (red foxes: 3.5 ± 2.2 km [0.6–7.4 km], *n* = 7; Arctic foxes: 1.6 ± 1.1 km [0.4–3.3 km], *n* = 4).

### 3.3 Excursions and commuting trips

In winter, all resident Arctic foxes used the sea ice, commuting at least once and up to seven times, although their commuting trips never lasted more than 3 days. However, no red foxes commuted to the sea ice. We found no overall difference in land excursion frequency between seasons or species (GLMM: t_species_ = −0.859, *p* = .397; t_season_ = −0.539, *p* = .593; t_season*species_ = 1.650, *p* = 0.109, *n* = 36). Weekly frequency of land excursion in red foxes increased from 0.05 [0–0.25] in summer to 0.13 [0–0.32] in winter, while Arctic fox land excursion frequency was 0.08 [0–0.21] in summer and 0.06 [0–0.13] in winter. Pooling together excursions and commuting trips, we found that these extraterritorial exploratory trips were more frequent in winter (GLMM: t_season_ = 3.113, *p* = 0.004, *n* = 36) but occurred at a similar frequency in both species (t_species_ = −1.547, *p* = 0.131). Table S6 provides all parameters from all GLMMs performed in this study.

## 4 DISCUSSION

Both red and Arctic foxes showed mixed movement tactics in our study area, some remaining resident and others engaging in long‐range movements, which denotes flexibility in both species’ spatial behavior. However, although resident red foxes used space similarly to resident Arctic foxes during summer, their winter strategy differed markedly. While home range or core area sizes did not differ between species during summer, when food is plentiful and the climate mild, red foxes substantially increased their ranging behavior in winter, whereas Arctic foxes did not. The harshness of winter abiotic conditions (i.e., duration of snow cover and low temperatures) is the main limitation to red fox distribution (Bartoń & Zalewski, 2007), while the availability of stable anthropogenic food sources was the main driver of their expansion across the Arctic (Gallant et al., 2020). Red foxes in the Arctic benefit from a high mass‐adjusted basal metabolic rate, which help them tolerate colder temperatures, but also increases their food requirements (Careau, Giroux, & Berteaux, 2007; Careau, Morand‐Ferron, & Thomas, 2007; Fuglesteg et al., 2006). This increase in food requirements occurs when food is scarce, most prey having migrated back South, and the rodents sheltered by a hard snow cover (Jędrzejewski & Jędrzejewska, 1992). Thus, this large seasonal increase in home range size likely reflected red foxes’ lack of adaptation to prey scarcity and the harsh conditions of the tundra during winter.

As predicted (P1), foxes did not disperse in summer while prey was abundant. During summer, foxes raising their young are constrained to remaining around breeding dens. However, the proportion of foxes raising a litter largely depends on spring resources (McDonald et al., 2017) and even in years when spring resources were at the lowest, no foxes dispersed the following summer. We also have indirect evidence that some foxes of this study did not breed (e.g., established in areas with no breeding dens, center of activity shifting often during summer), yet they still maintained a home range over the summer. The high dispersal rate in winter contrasted with the usually low proportion of dispersing adults found in both red and Arctic fox populations elsewhere (e.g., Lai et al., 2017; Storm et al., 1976; Walton et al., 2018), and other carnivores in general (e.g., Ferreras et al., 2004), suggesting that overwinter survival near the tree line was particularly difficult for both species. The higher dispersal rate in our study area could be due to low rodent densities compared to elsewhere, notably the Canadian High Arctic (Ehrich et al., 2020; Lai et al., 2022), and scarce access to anthropogenic subsidies unlike other areas of sympatry (Killengreen et al., 2011; Rød‐Eriksen et al., 2020). This high rate of dispersal could further indicate that foxes in our study area were less likely than other populations (such as Bylot Island Arctic foxes living near a snow goose colony that is much larger than those in Churchill) to capitalize on summer‐abundant resources, caching items to survive winters as residents (Rioux et al., 2017). In the case of Arctic foxes, it is also possible that the Hudson Bay sea ice is more productive than elsewhere (Lunn et al., 1997; Parks et al., 2006).

Dispersal can incur high fitness costs, with higher mortality or missed opportunities to reproduce following dispersal (e.g., Ferreras et al., 2004; Lai et al., 2017; Soulsbury et al., 2008). As expected, the survival cost of dispersal was high in our population, with 11 of 13 dispersers suffering mortality within 4 months of starting dispersal. Our results are consistent with observations from the Canadian High Arctic. While foxes of Bylot Island remained resident and were able to survive over multiple winters, on Herschel Island both red foxes dispersed and died (Lai et al., 2022). Mortality during dispersal may occur because dispersers must cross unfamiliar areas (e.g., Ferreras et al., 2004; Storm et al., 1976) and, in leaving our remote study area, may come into greater contact with humans (e.g., Ferreras et al., 2004). The cost of dispersal on reproduction was also likely high, as only three of our 13 dispersed foxes survived long enough through the subsequent breeding season to have successfully raised pups, whereas all but one of twelve residents survived long enough to raise pups successfully. However, remaining resident during prey scarcity may compromise reproduction too, as resources may be allocated to winter survival at the cost of next‐season reproduction (Löfgren et al., 1986). Although each tactic may have a cost, our results suggest that dispersing is risky for both red and Arctic foxes and may be a desperate tactic to cope with local prey scarcity.

Arctic foxes are well known for their long‐range movements, specifically using the sea ice (Fuglei & Tarroux, 2019; Lai et al., 2017; Pamperin et al., 2008; Tarroux et al., 2010). Two‐thirds of our Arctic foxes indeed dispersed using the sea ice as a platform, whereas red foxes never did. Instead, red foxes in our study dispersed inland, toward the boreal forest. The dispersal distances of red foxes, despite being shorter than those of Arctic foxes, were particularly large for this species. Only two studies have reported similar dispersal distances, one in Sweden and one in the Canadian High Arctic (Lai et al., 2022; Walton et al., 2018). The low cumulative to straight‐line dispersal distance ratio of these red foxes suggests straight relocation until finding suitable habitat. Arctic foxes, in contrast, seemed more prone to exploration during dispersal, suggesting they primarily use the sea ice for foraging (as suggested by diet studies; Roth, 2003), and not just as a dispersal platform. When rodent abundance is low, Arctic foxes respond numerically to marine resources, suggesting that exploiting the sea ice in winter is a well‐established strategy for responding to prey scarcity (Roth, 2003).

Further highlighting that sea ice is a key habitat for Arctic foxes and in partial agreement with P3, all Arctic foxes commuted to the sea ice. Anecdotally, one Arctic fox even had 76.7% of her winter home range on the sea ice, yet she still took five exploratory trips even farther onto the sea ice (Figure S1). Red foxes, however, never commuted to the sea ice, further suggesting they generally avoid this habitat, like on St. Matthews island where red foxes hunt inland while Arctic foxes used the coast (e.g., Klein & Sowls, 2015). Yet, direct and indirect evidence suggests that red foxes use sea ice occasionally, either to travel—red foxes are found on offshore islands sometimes quite far from the mainland, which suggest they use seasonal ice to disperse there (Andriashek et al., 1985; Klein & Sowls, 2015; Lai et al., 2022)—or to forage (Andriashek & Spencer, 1989; Jung et al., 2020)—interestingly, both reports of red foxes foraging on sea ice come from the same area in Yukon, Canada. Although these observations are not unique, they remain rare. While on Herschel Island (where winter food is scarce) a pair of red foxes left their summer home range and ventured on the sea ice (including intertidal ice), where they died relatively quickly (Lai et al., 2022), the red fox pair on Bylot Island (where food is less scarce) remained resident for multiple winters and never went to the sea ice, only relying on inland resources (Lai et al., 2022). On Herschel Island, red foxes were far from the boreal forest, while red foxes in our study area were close to the tree line, and thus had alternative habitats other than the sea ice. While sea ice may offer alternative resources when terrestrial prey is scarce, our red fox population did not exploit this habitat, likely due to their lack of adaptation to that particularly unpredictable and harsh environment (Klein & Sowls, 2015).

Red foxes’ difficulty to overwinter on the tundra is further reflected in the seasonal change in home range size of residents. This winter expansion of home range may originate from both a decrease in prey abundance and an increase in red foxes’ energetic requirements. Arctic foxes adapt to the harsh winter climate with a low resting metabolic rate (likely to conserve energy) and exceptional insulation (Fuglei & Øritsland, 1999; Fuglesteg et al., 2006; Prestrud, 1991). They also show metabolic depression (i.e., a significant decrease in the resting metabolic rate) in response to starvation, indicating an adaptation to food scarcity (Fuglei & Øritsland, 1999). Red foxes in the Arctic compensate for their poorer insulation with a higher basal metabolic rate, which benefits them by expanding their thermoneutral zone (Careau, Morand‐Ferron, & Thomas, 2007), but which also increases their energetic requirements (Fuglesteg et al., 2006). Yet, red foxes did not engage more often in dispersal or excursions than Arctic foxes, nor did their excursion rate clearly increase in winter (unlike P4 and P5). Expanding their home range during winter may have been sufficient for residents to obtain enough prey.

The obstinate strategy hypothesis states that animals may not adjust their ranging behavior to the fluctuation of resources because fighting competitors to expand a home range is costly (von Schantz, 1984). Our red fox population instead behaved as flexible strategists, unlike many other carnivore populations (Eide et al., 2004; López‐Bao et al., 2019; Meia & Weber, 1995). Winter home ranges of these red foxes averaged ~35 km^2^ (up to 56 km^2^), among the largest reported for this species (Goszczyński, 2002; Lai et al., 2022; Walton et al., 2017). Large home ranges suggest a low fox density in our area (Trewhella et al., 1988), which may decrease the cost of expanding the home range in winter, compared with maintaining such a large home range during summer. Anecdotally, one red fox captured on the tundra relocated to the boreal forest long enough during winter to calculate two core areas and home ranges (tundra and forest): Her forest home range was 25% the size of her tundra home range (only 14% for core areas), suggesting that forest habitat had higher prey density and milder abiotic conditions than the tundra.

We found low seasonal overlap of individual core areas and large distances between core area centroids, indicating relatively low site fidelity, and thus quite high spatial flexibility in both species. The flexible and the obstinate strategies are two ends of a continuum that depend on the amplitude of resource fluctuation and the species’ life span relative to the periodicity of resource fluctuation. In the Canadian High Arctic, Arctic foxes behaved as flexible strategists, unlike in the European Arctic (e.g., Eide et al., 2004), adjusting the size of their home range yearly to lemming density (Tarroux, 2011). Hyenas have also shown mixed strategies at the species level, some clans behaving as obstinate and other clans as flexible strategists in response to resource fluctuation (Maude et al., 2019). Such behavioral plasticity in carnivores may allow them to adapt to future changes in prey abundance and distribution linked to climate change (Nater et al., 2021).

Although current conditions of food scarcity during winter may limit red fox density (Gallant et al., 2012, 2020), resident red foxes were able to overwinter without relying on anthropogenic subsidies and they did not engage in risky dispersal more often than their congeners. The hindrance to overwinter survival imposed by their lack of adaptation to food scarcity and the harsh conditions of the tundra seem therefore limited at the tree line, where they may be able to use sparse forest patches to buffer the tundra's low food availability.

Our study generally supports the idea that movement strategies in both red and Arctic foxes are mostly driven by seasonal fluctuations of resources and that both species are highly flexible. Current winter conditions seem limiting to the Churchill red fox population: most individuals dispersed, and the residents needed to increase their home range to find enough resources to survive winter, suggesting that food scarcity during winter may limit red fox density (Gallant et al., 2012, 2020). However, Arctic regions are warming up to four times faster than the rest of the globe (You et al., 2021), and due to climate‐induced variability in environmental conditions, Arctic ecosystems are not at equilibrium. The red fox is among the most adaptable mammals (Wells & Aubry, 2011) and as such may adapt to new conditions and change its behavior in the future.

Arctic foxes foraged on the sea ice instead of expanding their home ranges. Sea‐ice‐dependent predators may lose opportunities to cope with terrestrial food scarcity, as sea ice will be negatively impacted as the Arctic warms. However, although the negative impact of Arctic warming on most native Arctic wildlife is widely recognized (e.g., Descamps et al., 2017; Molnár et al., 2010; Post et al., 2009), the direction of these changes may be more difficult to predict for expanding species. Some effects may benefit these boreal‐forest species. For example, milder winters may lower the costs associated with thermoregulation (Nater et al., 2021; Pálsson et al., 2016), and changing tundra communities will provide expanding species with increased foraging opportunities (Post et al., 2009; Tape et al., 2016), favoring boreal‐forest species’ persistence in this environment (Callaghan et al., 2004). The species interaction‐abiotic stress hypothesis indeed proposes that abiotic stress mostly limits a species’ distribution in areas where climate imposes stressful conditions (e.g., cold edge of a species’ range), while interactions with heterospecific competitors likely limit species distributions in milder areas (e.g., Louthan et al., 2015). However, climate‐induced changes in the Arctic are also having dire consequences (such as rain‐on‐snow events and melt‐freeze cycles) on the persistence of many herbivorous species (Berteaux et al., 2017; Forbes et al., 2016; Stien et al., 2010). For example, such dramatic declines in some crucial prey may reach critical winter thresholds that trigger important dispersal in highly mobile predators, or prevent newcomers from becoming established, which could lead to local extirpation of both expanding and native predator species. In a warming Arctic, we propose that both leading‐ and trailing‐edge predator populations may, thus, also become directly limited by climate‐induced declines in prey availability. The persistence of expanding populations and the outcome of their competition with tundra‐native species will likely vary greatly spatiotemporally based on current local conditions.

## AUTHOR CONTRIBUTIONS

**Chloé Warret Rodrigues:** Conceptualization (equal); data curation (lead); formal analysis (lead); investigation (lead); methodology (lead); project administration (equal); software (lead); supervision (lead); validation (lead); visualization (lead); writing – original draft (lead); writing – review and editing (equal). **James Roth:** Conceptualization (equal); funding acquisition (lead); investigation (supporting); project administration (equal); resources (lead); supervision (supporting); validation (supporting); visualization (supporting); writing – review and editing (equal).

## CONFLICT OF INTEREST STATEMENT

We have no conflicts of interest to disclose.

## Supporting information


Figure S1.

Data S1.

Supplemental File S1.

## Data Availability

The raw tracking data associated with this analysis are stored on the Dryad Digital Repository: https://doi.org/10.5061/dryad.r7sqv9shp.
