# Supplementary figures and images for: Feast to famine: Sympatric predators respond differently to seasonal prey scarcity on the low Arctic tundra
Source: Ecol Evol. 2023 Mar 27;13(3):e9951. doi: 10.1002/ece3.9951 (PMC10041551; doi:10.1002/ece3.9951)

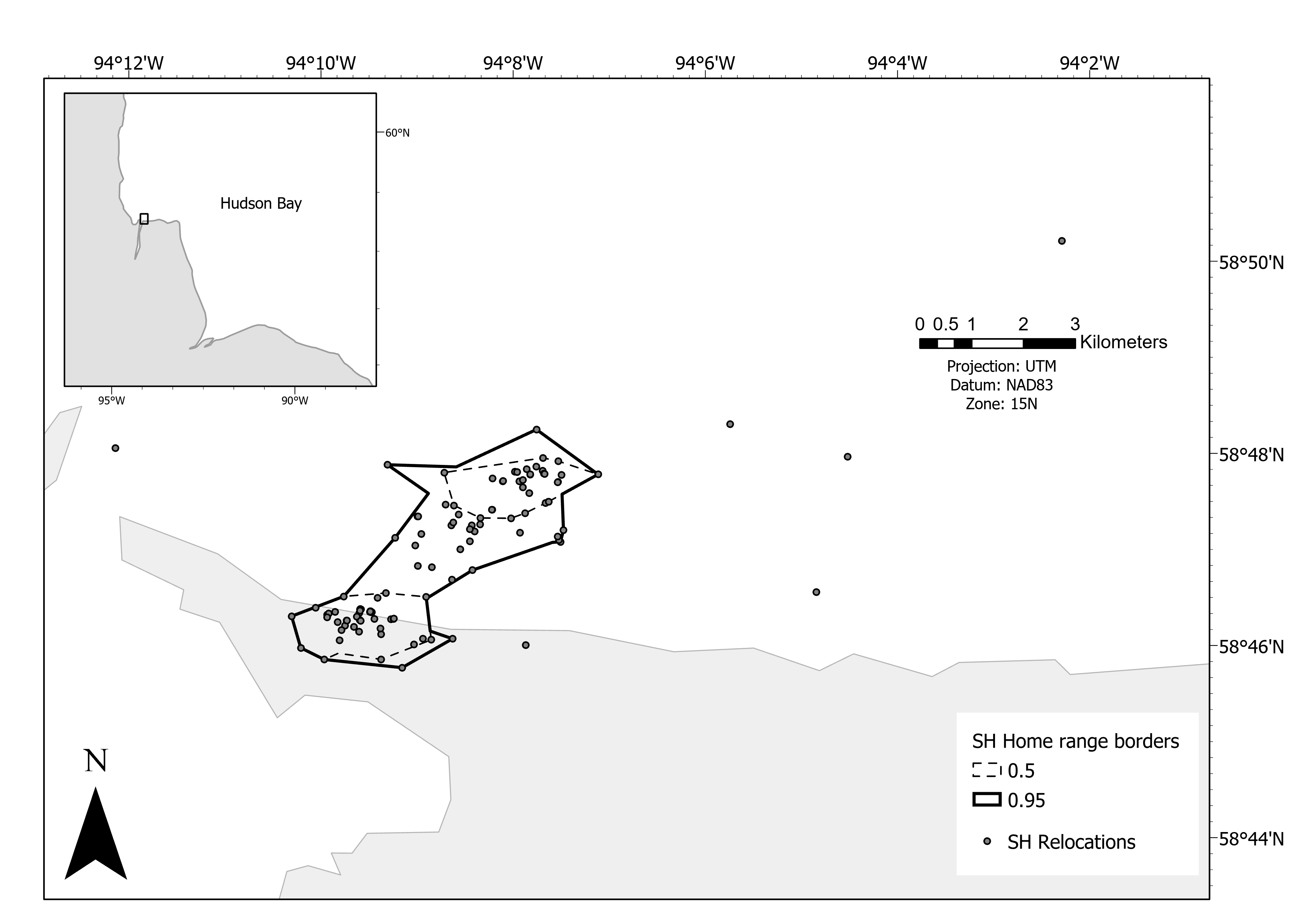

Supplement: Supplementary file 1 — Figure S1. [file ECE3-13-e9951-s001.png]
