# Supplementary material for: Feast to famine: Sympatric predators respond differently to seasonal prey scarcity on the low Arctic tundra
Source: Ecol Evol. 2023 Mar 27;13(3):e9951. doi: 10.1002/ece3.9951 (PMC10041551; doi:10.1002/ece3.9951)
Supplement: Supplementary file 2 — Data S1. [file ECE3-13-e9951-s002.docx]

**Supplementary data – Feast to famine: sympatric predators respond differently to seasonal prey scarcity on the low-Arctic tundra**

**Table S1.** Fix schedule of our different fox collars (purchased in 2 batches).

| Number of collars | Year | Dates | Update period (hours) |
| --- | --- | --- | --- |
| 5 | 2017 | 10 April-30 June | 2 |
| 5 | 2017 | 1 July-30 August | 4 |
| 5 | 2017 | 1 September-31 December | 24 |
| 5 | 2018 | 1 January-9 April | 24 |
| 3 | 2018 | 10 April-30 June | 2 |
| 2 | 2018 | 1 July-30 August | 4 |
| 2 | 2018 | 1 September-31 December | 24 |
| 1 | 2019 | 1 January-9 April | 24 |
| 5 | 2018 | 10 April-14 June | 1.5 |
| 4 | 2018 | 15 June-14 August | 6 |
| 4 | 2018 | 15 August-14 December | 24 |
| 2 | 2018 | 15 December-31 December | 6 |
| 2 | 2019 | 1 January-14 March | 6 |
| 13 | 2019 | 15 March-14 June | 1.5 |
| 10 | 2019 | 15 June-30 September | 6 |
| 9 | 2019 | 1 October-14 December | 24 |
| 7 | 2019 | 15 December-31 December | 6 |
| 6 | 2020 | 1 January-14 March | 6 |
| 6 | 2020 | 15 March-14 June | 1.5 |
| 4 | 2020 | 15 June-30 September | 6 |
| 1 | 2020 | 1 October-31 December | 24 |
| 1 | 2021 | 1 January-3 February* | 24 |

* The last collar’s battery died on February 3, 2021.

**Table S2.** Winter (Nov. 1^st^ – May 14) and summer (May 15 – Oct. 31^st^) home-range areas of individuals fitted with Telonics satellite collars in northern Manitoba between 2017 and 2020. Home ranges were estimated based on one location per day using an *a*-Local Convex Hull (LoCoH) and a classic bivariate kernel density estimator (KDE) with reference bandwidth. LoCoH estimates were used in this study, and KDE are provided for cross-study comparison.

| **Fox ID** | **species** | **sex** | **Period** | **Area UD 95%** | |
| --- | --- | --- | --- | --- | --- |
|  |  |  |  | **LoCoH** | **KDE** |
| MM | Arctic fox | M | summer 2017 | 16.48 | 47.20 |
| U | Arctic fox | M | summer 2017 | 15.35 | 25.99 |
| GK | Arctic fox | M | summer 2018 | 6.30 | 13.19 |
| MJ | Arctic fox | F | summer 2018 | 26.67 | 63.67 |
| MM | Arctic fox | M | summer 2018 | 4.37 | 9.69 |
| SH | Arctic fox | F | summer 2018 | 0.94 | 31.52 |
| B | Arctic fox | F | summer 2019 | 20.82 | 38.00 |
| DL | Arctic fox | M | summer 2019 | 12.17 | 32.53 |
| M | Arctic fox | M | summer 2019 | 33.91 | 71.93 |
| SH | Arctic fox | F | summer 2019 | 22.06 | 75.14 |
| T | Arctic fox | F | summer 2019 | 18.79 | 33.59 |
| T | Arctic fox | F | summer 2020 | 14.99 | 155.95 |
| AB | Red fox | F | summer 2017 | 17.08 | 35.94 |
| FJ | Red fox | M | summer 2017 | 28.61 | 188.41 |
| LR | Red fox | M | summer 2017 | 15.49 | 34.00 |
| AB | Red fox | F | summer 2018 | 16.54 | 51.04 |
| LB | Red fox | F | summer 2018 | 10.30 | 39.30 |
| A | Red fox | F | summer 2019 | 24.04 | 78.32 |
| I | Red fox | F | summer 2019 | 12.44 | 54.70 |
| LR | Red fox | M | summer 2019 | 16.76 | 57.96 |
| S | Red fox | F | summer 2019 | 9.84 | 31.69 |
| A | Red fox | F | summer 2020 | 27.08 | 60.72 |
| I | Red fox | F | summer 2020 | 16.67 | 54.43 |
| LR | Red fox | M | summer 2020 | 21.88 | 57.69 |
| MM | Arctic fox | M | winter 2018 | 19.86 | 90.19 |
| U | Arctic fox | M | winter 2018 | 9.94 | 32.79 |
| SH | Arctic fox | F | winter 2019 | 5.65 | 33.45 |
| DL | Arctic fox | M | winter 2020 | 16.35 | 58.89 |
| M | Arctic fox | M | winter 2020 | 44.32 | 305.39 |
| AB | Red fox | F | winter 2018 | 31.56 | 61.70 |
| LR | Red fox | M | winter 2018 | 31.76 | 78.85 |
| AB | Red fox | F | winter 2019 | 22.13 | 101.99 |
| A | Red fox | F | winter 2020 | 56.58 | 104.77 |
| I | Red fox | F | winter 2020 | 33.90 | 63.50 |
| LR | Red fox | M | winter 2020 | 40.08 | 78.98 |
| S | Red fox | F | winter 2020 | 24.43 | 89.46 |

**Table S3.** Average number of hours between relocations we used to build each home range of the resident red and Arctic foxes collared in northern Manitoba, with standard error (SE), range (min and max), and sample size (n) after thinning the tracks to keep 1 location per day.

| **Fox ID** | **year** | **season** | **mean** | **se** | **min** | **max** | **n** |
| --- | --- | --- | --- | --- | --- | --- | --- |
| AB | 2017 | summer | 25.88 | 0.88 | 2 | 96 | 156 |
| AB | 2018 | summer | 28.24 | 1.45 | 24 | 144 | 113 |
| A | 2019 | summer | 24.55 | 0.75 | 6 | 72 | 165 |
| A | 2020 | summer | 23.8 | 1.12 | 5 | 42 | 74 |
| B | 2019 | summer | 27.1 | 1.29 | 6 | 168 | 149 |
| DL | 2019 | summer | 24.12 | 0.7 | 5 | 48 | 168 |
| FJ | 2017 | summer | 24.49 | 0.66 | 4 | 48 | 165 |
| GK | 2018 | summer | 24.98 | 0.64 | 6 | 48 | 162 |
| I | 2019 | summer | 24.45 | 0.69 | 6 | 53 | 165 |
| I | 2020 | summer | 24.78 | 1.1 | 6 | 48 | 83 |
| LR | 2017 | summer | 25.34 | 0.74 | 2 | 48 | 160 |
| LR | 2019 | summer | 32.27 | 2.46 | 12 | 240 | 125 |
| LR | 2020 | summer | 33.82 | 3.29 | 11 | 144 | 70 |
| LB | 2018 | summer | 25.08 | 0.67 | 6 | 48 | 104 |
| MM | 2017 | summer | 30.23 | 2.8 | 4 | 72 | 133 |
| MM | 2018 | summer | 27.59 | 1.13 | 23 | 72 | 81 |
| MJ | 2018 | summer | 27.07 | 1.41 | 2 | 78 | 91 |
| M | 2019 | summer | 25.79 | 0.99 | 6 | 96 | 151 |
| SH | 2018 | summer | 28.25 | 3.44 | 6 | 312 | 87 |
| SH | 2019 | summer | 23.89 | 0.76 | 6 | 42 | 168 |
| S | 2019 | summer | 24.39 | 0.72 | 6 | 48 | 166 |
| T | 2019 | summer | 25.49 | 0.89 | 6 | 102 | 158 |
| T | 2020 | summer | 25.7 | 1.35 | 2 | 66 | 69 |
| U | 2017 | summer | 25.49 | 0.81 | 4 | 72 | 159 |
| AB | 2018 | winter | 25.71 | 0.98 | 23 | 168 | 182 |
| AB | 2019 | winter | 29.17 | 2.71 | 24 | 168 | 65 |
| A | 2020 | winter | 25.19 | 0.83 | 2 | 84 | 186 |
| DL | 2020 | winter | 23.63 | 0.68 | 6 | 36 | 60 |
| FJ | 2018 | winter | 26.11 | 0.91 | 24 | 48 | 57 |
| GK | 2018 | winter | 26.53 | 2.53 | 24 | 120 | 38 |
| I | 2020 | winter | 24.34 | 0.62 | 6 | 66 | 193 |
| LR | 2018 | winter | 25.48 | 0.78 | 8 | 104 | 183 |
| LR | 2020 | winter | 29.14 | 1.35 | 2 | 144 | 161 |
| MM | 2018 | winter | 25.3 | 0.55 | 23 | 96 | 184 |
| M | 2020 | winter | 24.85 | 0.93 | 6 | 119 | 139 |
| SH | 2019 | winter | 23.81 | 0.76 | 6 | 42 | 113 |
| S | 2020 | winter | 28.83 | 2.4 | 6 | 144 | 65 |
| U | 2018 | winter | 24.35 | 0.35 | 24 | 48 | 68 |

**Table S4.** History of each Arctic fox (AF) and red fox (RF) captured in northern Manitoba.

| **Fox ID** | **species** | **track start date** | **track end date** | **Fate** | **winter home-range analysis** | **summer home-range analysis** | **dispersal analysis** |
| --- | --- | --- | --- | --- | --- | --- | --- |
| U | AF | 2017-05-08 | 2018-02-06 | died on sea ice | 1 | 1 | 1 |
| MM | AF | 2017-05-09 | 2018-10-07 | died on land (at den) | 1 | 2 | 0 |
| GH | AF | 2018-04-18 | 2018-05-21 | died on intertidal ice | 0 | 0 | 0 |
| GI | AF | 2018-04-15 | 2018-06-03 | died on land (at den) | 0 | 0 | 0 |
| MJ | AF | 2018-04-20 | 2018-09-22 | died on land | 0 | 1 | 1 |
| GK | AF | 2018-04-21 | 2018-12-13 | died on land | 0 | 1 | 1 |
| SH | AF | 2018-06-20 | 2019-11-20 | battery expired | 1 | 2 | 2 |
| CN | AF | 2019-04-07 | 2019-05-04 | died on land | 0 | 0 | 1 |
| B | AF | 2019-04-09 | 2019-11-23 | died on land (at den) | 0 | 1 | 0 |
| MP | AF | 2019-04-09 | 2019-07-08 | died on land | 0 | 0 | 1 |
| M | AF | 2019-04-17 | 2020-03-22 | died on land (harvested) | 1 | 1 | 1 |
| DL | AF | 2019-04-30 | 2019-12-30 | died on sea ice | 1 | 1 | 0 |
| T | AF | 2019-05-02 | 2020-07-30 | battery expired | 0 | 2 | 1 |
| LR | RF | 2017-05-08 | 2018-05-28 | unit damaged | 1 | 1 | 0 |
| FJ | RF | 2017-05-09 | 2018-02-01 | died on land | 0 | 1 | 1 |
| AB | RF | 2017-05-09 | 2019-01-21 | died on land (harvested) | 2 | 2 | 1 |
| W | RF | 2018-04-18 | 2018-06-07 | malfunction | 0 | 0 | 0 |
| LB | RF | 2018-07-15 | 2019-02-08 | died on land (harvested) | 0 | 1 | 1 |
| BG | RF | 2019-03-20 | 2019-04-12 | died on land | 0 | 0 | 1 |
| BR | RF | 2019-04-06 | 2019-05-23 | died on land (at den) | 0 | 0 | 0 |
| LR* | RF | 2019-04-09 | 2021-02-03 | battery expired | 1 | 2 | 0 |
| A | RF | 2019-04-09 | 2020-07-29 | battery expired | 1 | 2 | 0 |
| S | RF | 2019-04-09 | 2020-04-18 | died on land | 1 | 1 | 1 |
| I | RF | 2019-04-10 | 2020-08-10 | battery expired | 1 | 2 | 0 |

*This fox was captured a second time and fitted with a new collar

**Table S5.** Detailed parameters of the dispersal tracks of Arctic (AF) and red (RF) foxes that left their home range (on start date) and their fate.

| Fox ID | species | start date (d/m/y) | end date (d/m/y) | duration (d) | total distance (km) | speed (km/d) | track direction (deg. from N) | habitat used for dispersal | survival time after dispersal (d) | survived into next reproductive season |
| --- | --- | --- | --- | --- | --- | --- | --- | --- | --- | --- |
| SH | AF | 15-Sep-18 | 8-Nov-18 | 54 | 781.73 | 14.48 | 183.5 | land | > 377 | Yes |
| SH | AF | 3-Mar-19 | 2-May-19 | 60 | 2133.31 | 35.56 | 77.8 | sea ice | > 202 | Yes |
| U | AF | 9-Jan-18 | 4-Feb-18 | 26 | 841.55 | 32.37 | 81.0 | sea ice | 0 | No |
| MJ | AF | 1-May-18 | 20-May-18 | 19 | 426.35 | 22.44 | 324.9 | land | 125 | Yes |
| MP | AF | 10-Apr-19 | 24-May-19 | 44 | 423.86 | 9.63 | 338.2 | land | 45 | Yes |
| GK | AF | 14-Nov-18 | 13-Dec-18 | 29 | 832.05 | 28.69 | 115.6 | both | 0 | No |
| CN* | AF | 8-Apr-19 | 23-Apr-19 | 15 | 216.30 | 14.42 | 258.9 | both | 11 | No |
| T | AF | 18-Nov-19 | 1-Apr-20 | 135 | 5197.27 | 38.50 | 79.5 | sea ice | > 120 | Yes |
| M | AF | 23-Feb-20 | 29-Feb-20 | 6 | 333.45 | 55.58 | 353.1 | both | 18 | No |
| FJ | RF | 3-Jan-18 | 13-Jan-18 | 10 | 280.62 | 28.06 | 294.0 | land | 19 | No |
| AB | RF | 26-Dec-18 | 18-Jan-19 | 23 | 167.92 | 7.30 | 208.5 | land | 0 | No |
| LB | RF | 24-Nov-18 | 15-Dec-18 | 21 | 259.92 | 12.38 | 193.3 | land | 55 | No |
| BG* | RF | 28-Mar-19 | 12-Apr-19 | 15 | 144.81 | 9.65 | 185.3 | land | 0 | No |
| S | RF | 20-Jan-20 | 28-Jan-20 | 8 | 150.58 | 18.82 | 210.3 | land | 81 | No |

* Fox was captured while dispersing. All parameters were thus estimated from the point and date of capture.

**measured in June of the year the dispersal started.

**Table S6.** Parameters (β coefficient, 95% confidence interval [2.50%-97.50%], df = degree of freedom, Z or t ratio, associated p-value and n = sample size) of all GLMMs to fit the home range and core area size of red and Arctic foxes in northern Manitoba, Canada.

|  | **Covariate** | **Estimate** | **Confidence intervals** | | **df** | **Z or t ratio** | **P value** |
| --- | --- | --- | --- | --- | --- | --- | --- |
| **model** |  |  | **2.50%** | **97.50%** |  |  |  |
| UD95 ~ Species*Season + Species + Season + (1\|fox ID) | Intercept | 16.93 | 11.55 | 23.05 | 16.97 | 5.170 | < 0.001 |
| n = 36 | Species | 0.69 | -8.95 | 9.73 | 15.78 | 0.143 | 0.888 |
| (Random effect variance±SD = 63.45±7.97) | Season | 2.65 | -4.40 | 9.79 | 20.46 | 0.718 | 0.481 |
|  | Species*Season | 14.88 | 5.11 | 25.52 | 19.84 | 3.060 | < 0.001 |
| Dispersal ~ Species + (1\|fox ID) | Intercept | 0.92 | -0.66 | 14.66 |  | 1.095 | 0.273 |
| n = 16 | Species | -1.14 | -23.92 | 2.93 |  | -1.063 | 0.288 |
| (Random effect variance = 0)^a^ |  |  |  |  |  |  |  |
| land excursions ~ Species*Season + (1\|fox ID) | Intercept | 0.08 | 0.03 | 0.14 | 32.00 | 3.135 | 0.004 |
| n = 36 | Species | -0.03 | -0.12 | 0.05 | 32.00 | -0.859 | 0.397 |
| (Random effect variance = 0)^a^ | Season | -0.03 | -0.14 | 0.07 | 32.00 | -0.539 | 0.594 |
|  | Species*Season | 0.11 | -0.02 | 0.25 | 32.00 | 1.650 | 0.109 |

^a^Excluding the random effect from these two models does not change any of the parameters. We chose to retain the random effect based on philosophical grounds (Bolker et al. 2009; Pasch et al. 2013).

**References**

Bolker, B.M., Brooks, M.E., Clark, C.J., Geange, S.W., Poulsen, J.R., Stevens, M.H.H., and White, J.S.S. 2009. Generalized linear mixed models: a practical guide for ecology and evolution. Trends Ecol. Evol. **24**(3): 127–135. doi:10.1016/j.tree.2008.10.008.

Pasch, B., Bolker, B.M., and Phelps, S.M. 2013. Interspecific dominance via vocal interactions mediates altitudinal zonation in neotropical singing mice. Am. Nat. **182**(5): 161–173. doi:10.1086/673263.


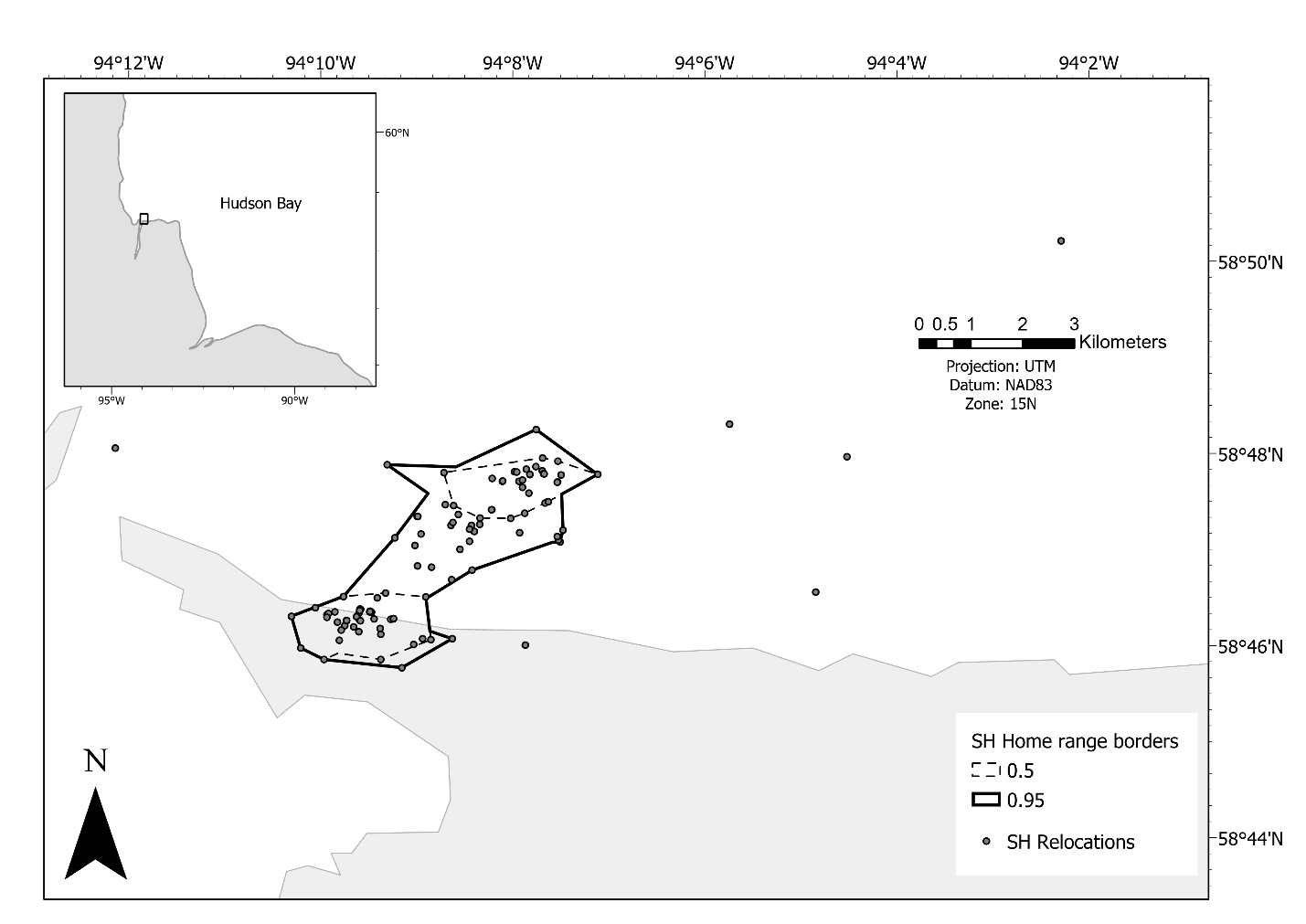


**Fig. S1.** Relocations, home range (UD95) and core area (UD50) of the Arctic fox SH in winter 2019. Ice (sea and Churchill River estuary) is in white and land in grey.
