# Supplementary material for: Feast to famine: Sympatric predators respond differently to seasonal prey scarcity on the low Arctic tundra
Source: Ecol Evol. 2023 Mar 27;13(3):e9951. doi: 10.1002/ece3.9951 (PMC10041551; doi:10.1002/ece3.9951)
Supplement: Supplementary file 3 — Supplemental File S1. [file ECE3-13-e9951-s003.pdf]

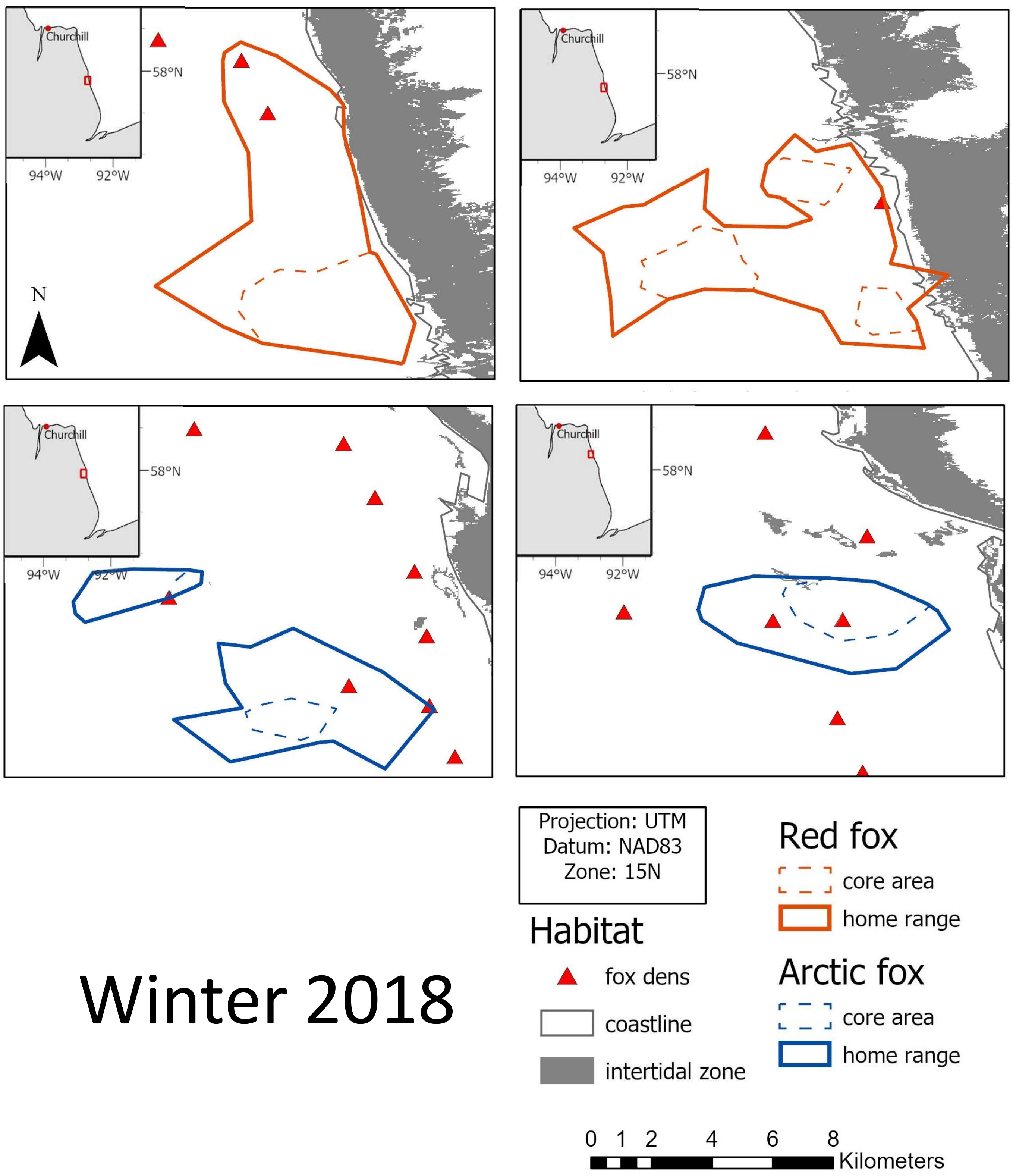
Churchill
58°N
94°W
92°W
N
Projection: UTM
Datum: NAD83
Zone: 15N
Habitat
fox dens
coastline
intertidal zone
Red fox
core area
home range
Arctic fox
core area
home range
Winter 2018
0 1 2 4 6 8 Kilometers

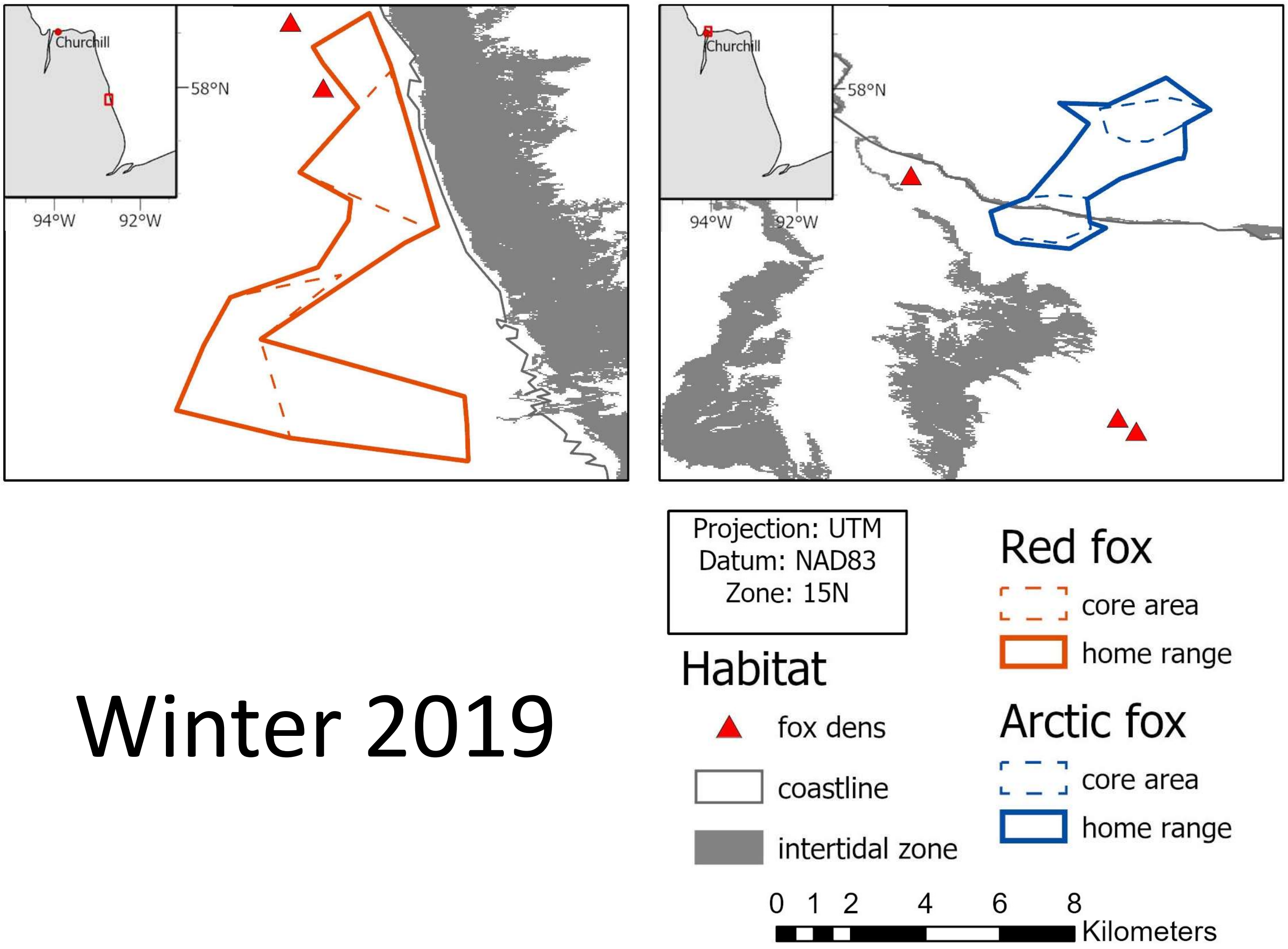

Churchill
58°N
94°W
92°W
Churchill
58°N
94°W
92°W
Projection: UTM
Datum: NAD83
Zone: 15N
Habitat
fox dens
coastline
intertidal zone
Red fox
core area
home range
Arctic fox
core area
home range
0 1 2 4 6 8
Kilometers

Winter 2019

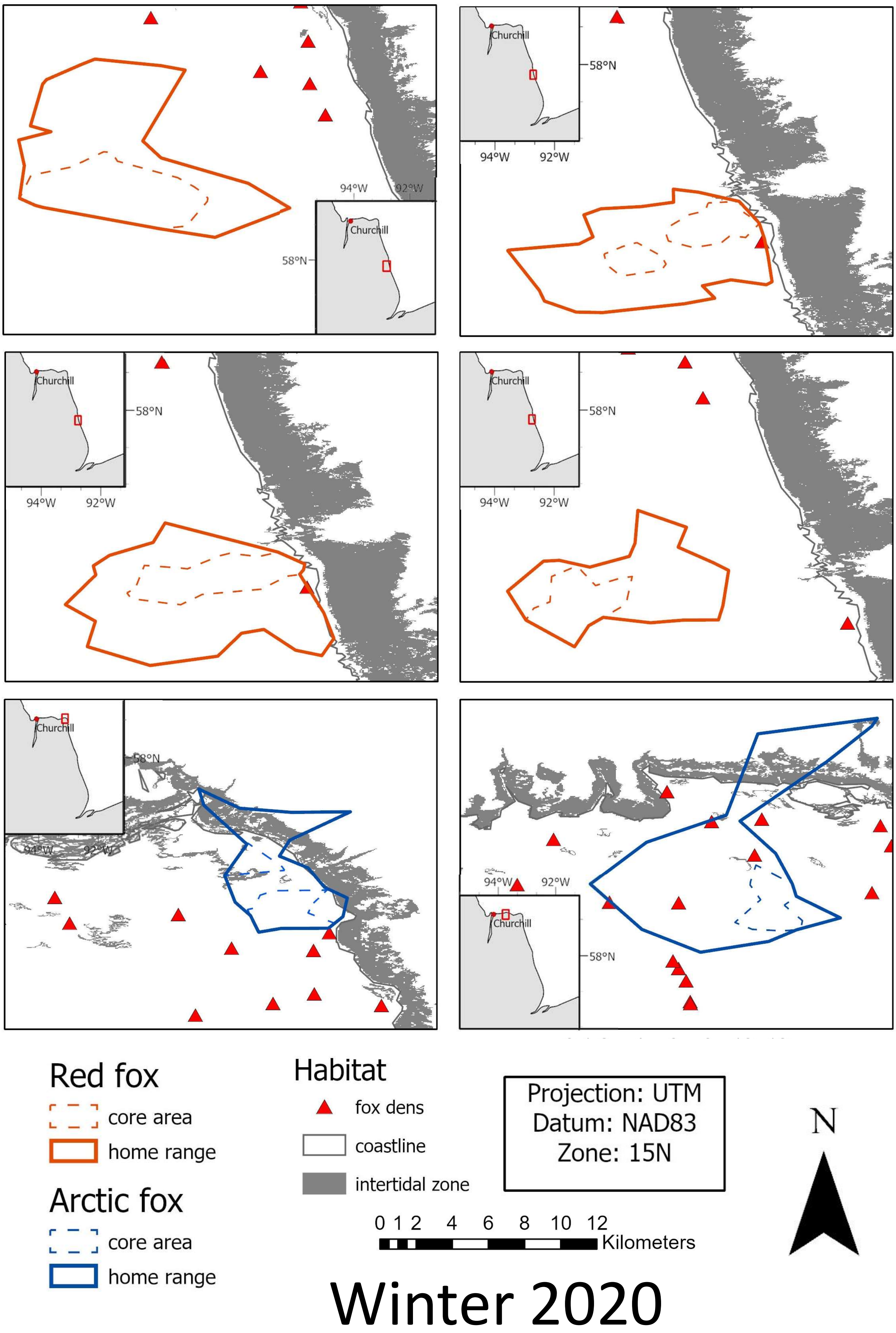
Churchill
58°N
94°W
92°W
Red fox
core area
home range
Arctic fox
core area
home range
Habitat
fox dens
coastline
intertidal zone
Projection: UTM
Datum: NAD83
Zone: 15N
0 1 2 4 6 8 10 12
Kilometers
N
Winter 2020

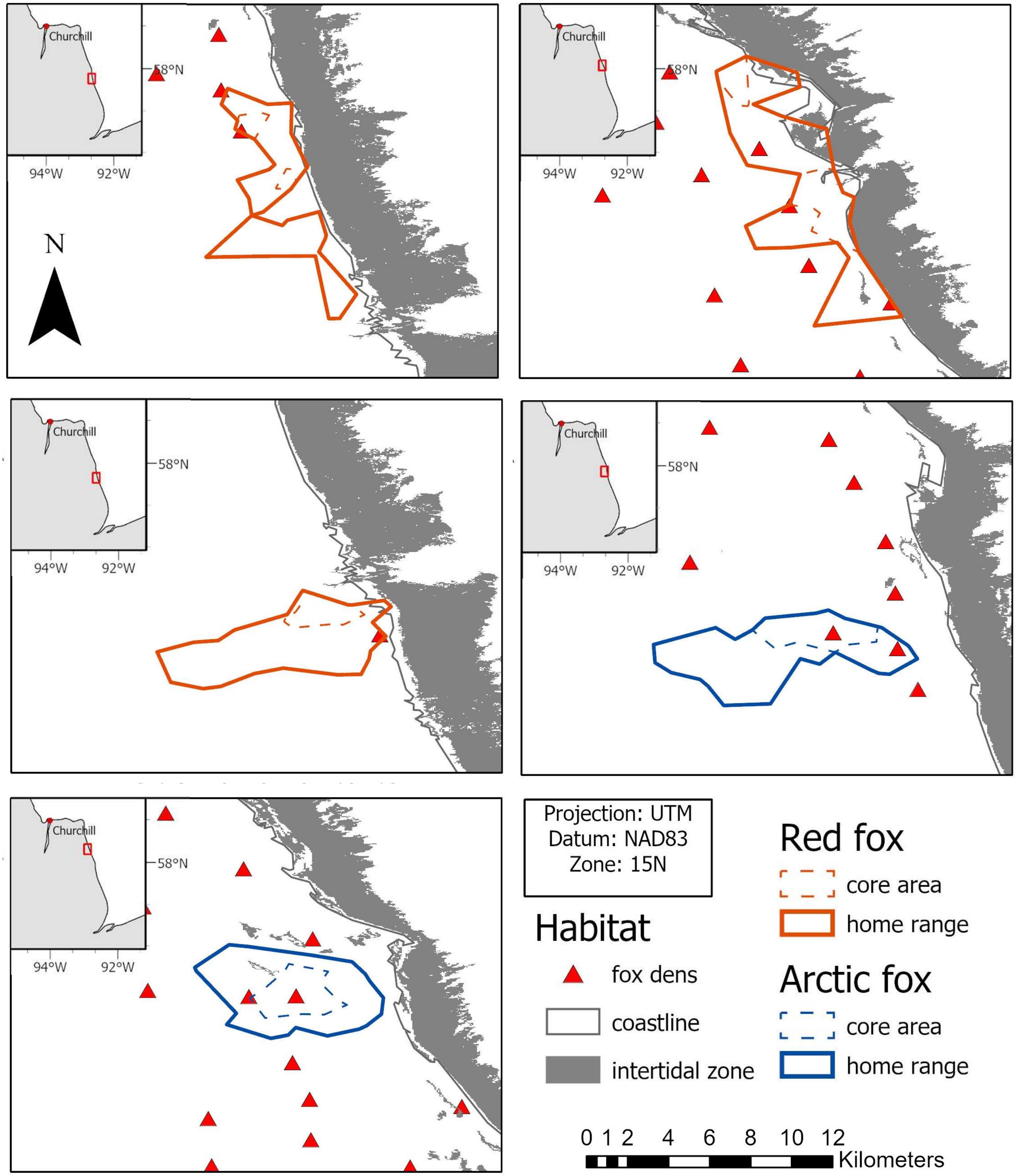
Churchill
58°N
94°W
92°W
N
Projection: UTM
Datum: NAD83
Zone: 15N
Habitat
fox dens
coastline
intertidal zone
Red fox
core area
home range
Arctic fox
core area
home range
0 1 2 4 6 8 10 12
Kilometers

Summer 2017

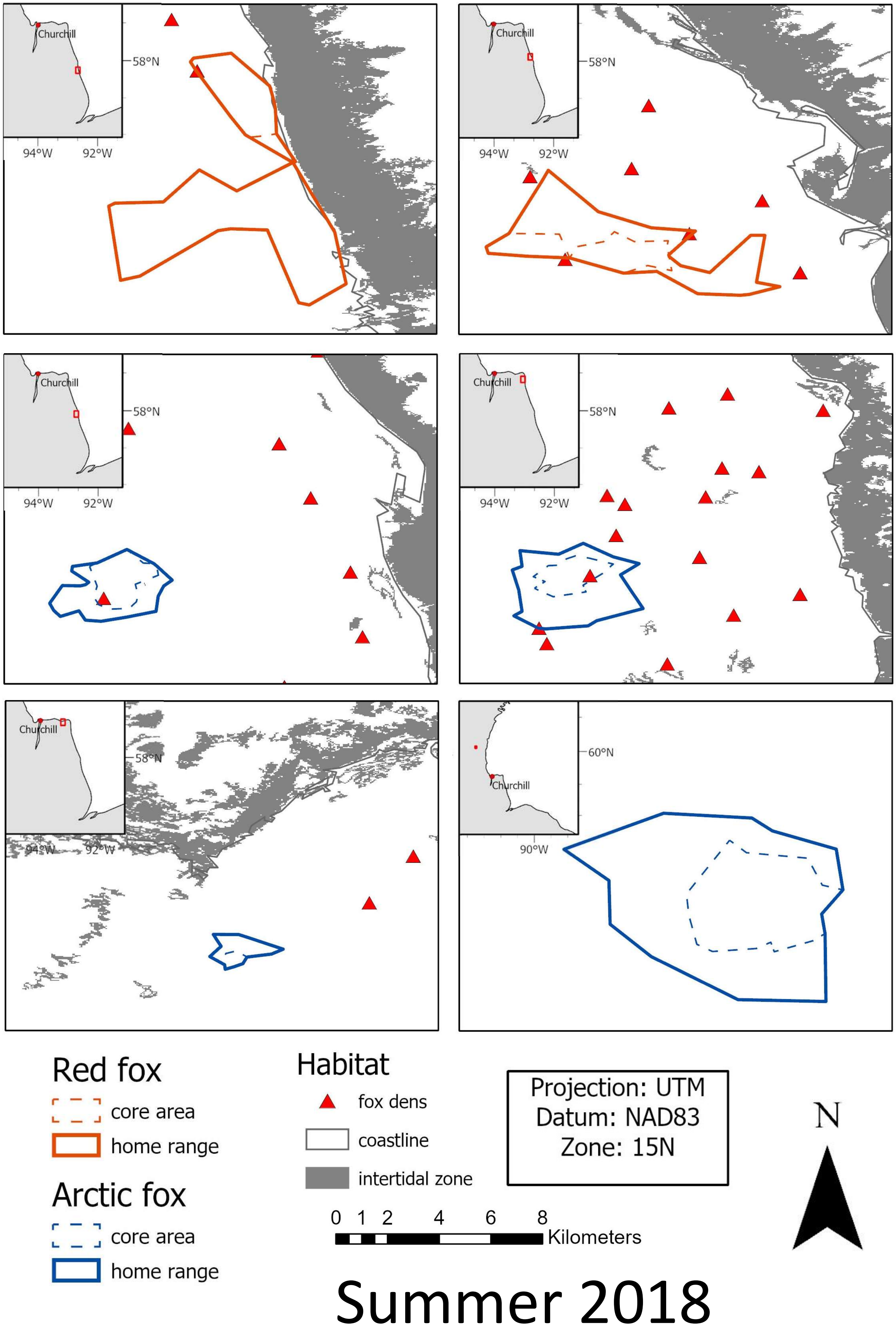
Churchill
58°N
94°W
92°W
60°N
90°W
Red fox
core area
home range
Arctic fox
core area
home range
Habitat
fox dens
coastline
intertidal zone
Projection: UTM
Datum: NAD83
Zone: 15N
0 1 2 4 6 8
Kilometers
N
Summer 2018

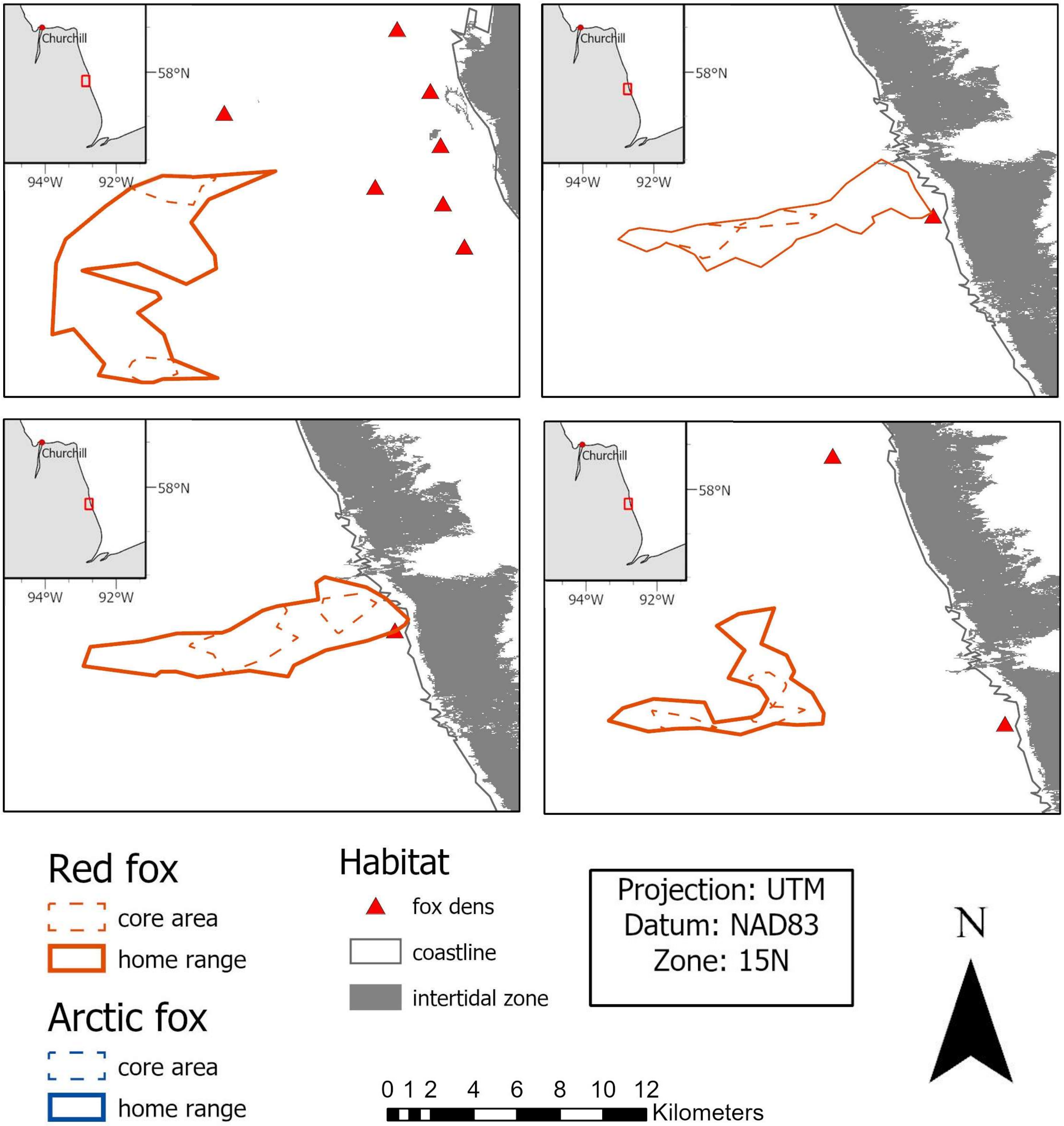
Churchill
58°N
94°W
92°W
Red fox
core area
home range
Arctic fox
core area
home range
Habitat
fox dens
coastline
intertidal zone
Projection: UTM
Datum: NAD83
Zone: 15N
N
0 1 2 4 6 8 10 12
Kilometers

Summer 2019

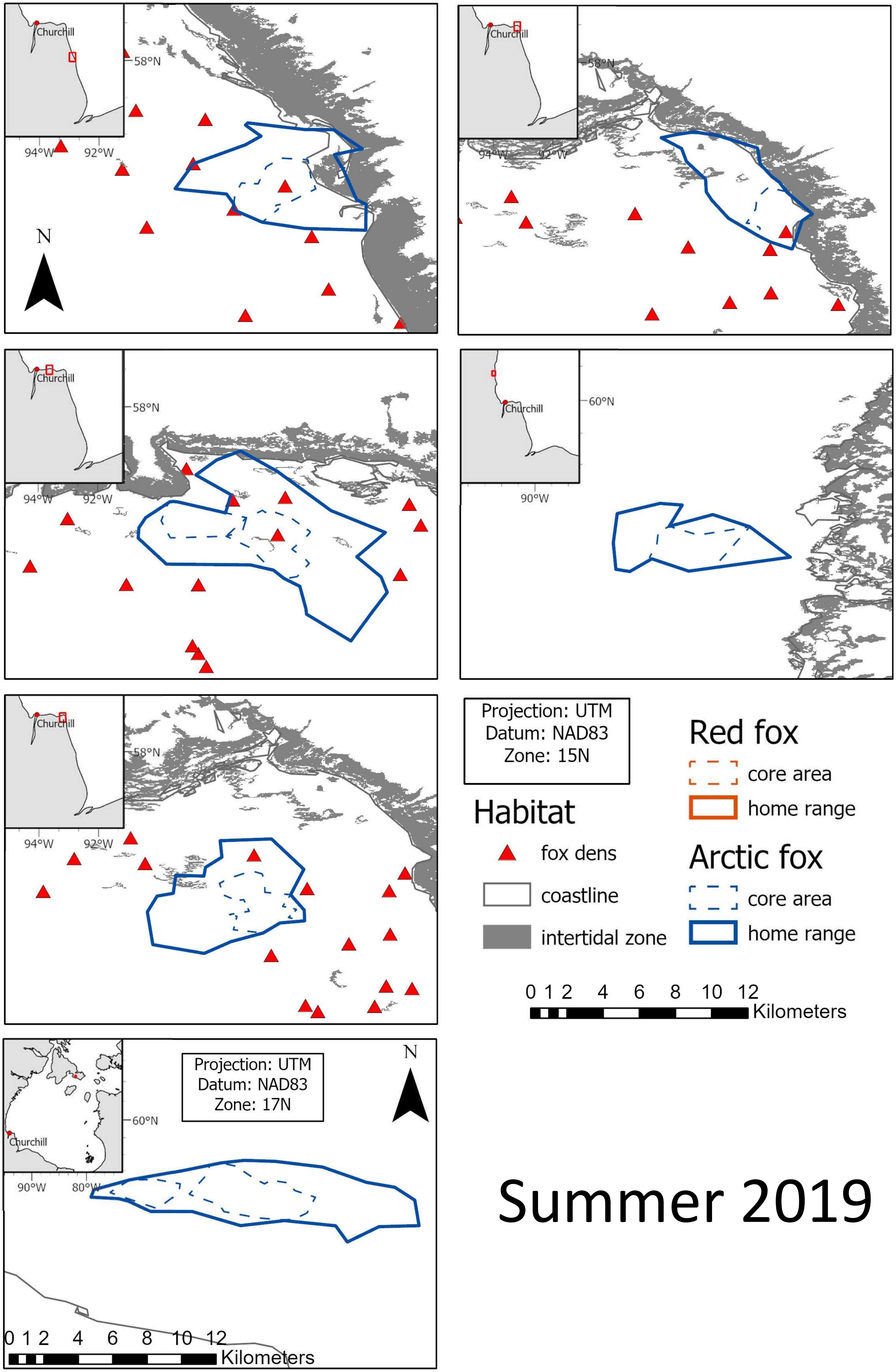
Churchill
58°N
94°W
92°W
90°W
80°W
60°N
N
Projection: UTM
Datum: NAD83
Zone: 15N
Habitat
fox dens
coastline
intertidal zone
Red fox
core area
home range
Arctic fox
core area
home range
0 1 2 4 6 8 10 12
Kilometers
Projection: UTM
Datum: NAD83
Zone: 17N
Summer 2019

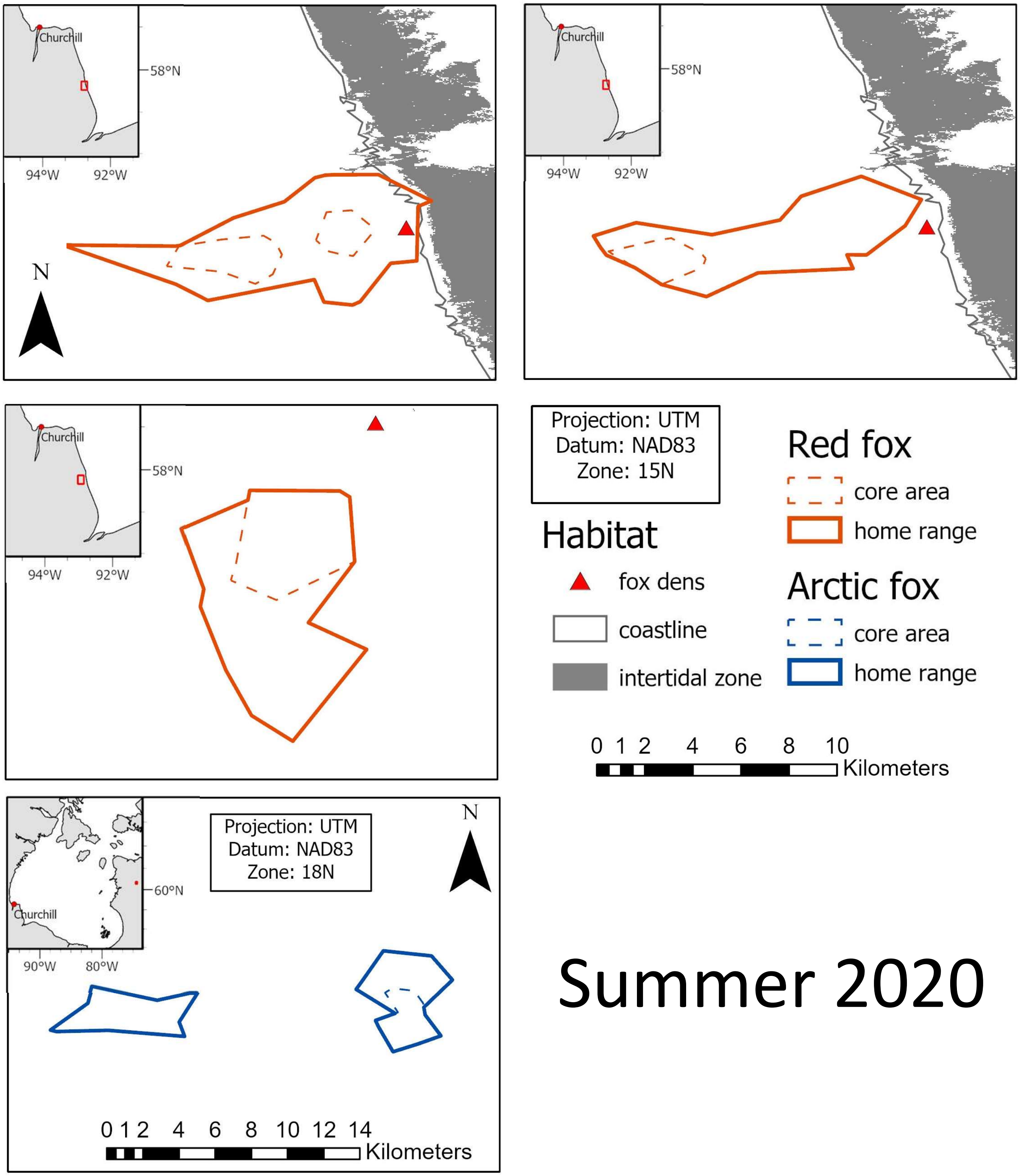
Churchill
58°N
94°W
92°W
N
Projection: UTM
Datum: NAD83
Zone: 15N
Habitat
fox dens
coastline
intertidal zone
Red fox
core area
home range
Arctic fox
core area
home range
0 1 2 4 6 8 10
Kilometers
Projection: UTM
Datum: NAD83
Zone: 18N
60°N
90°W
80°W
0 1 2 4 6 8 10 12 14
Kilometers
Summer 2020
